# Structure-Guided Redesign Improves NFL HIV Env Trimer Integrity and Identifies an Inter-Protomer Disulfide Permitting Post-Expression Cleavage

**DOI:** 10.3389/fimmu.2018.01631

**Published:** 2018-07-17

**Authors:** Lifei Yang, Shailendra Kumar Sharma, Christopher Cottrell, Javier Guenaga, Karen Tran, Richard Wilson, Anna-Janina Behrens, Max Crispin, Natalia de Val, Richard T. Wyatt

**Affiliations:** ^1^Department of Immunology and Microbiology, The Scripps Research Institute, La Jolla, CA, United States; ^2^International AIDS Vaccine Initiative, Neutralizing Antibody Center, The Scripps Research Institute, La Jolla, CA, United States; ^3^Department of Biochemistry, Oxford Glycobiology Institute, University of Oxford, Oxford, United Kingdom; ^4^Centre for Biological Sciences, Institute of Life Sciences, Highfield Campus, University of Southampton, Southampton, United Kingdom; ^5^Center for Molecular Microscopy (CMM), Center for Cancer Research (CCR), National Cancer Institute (NCI), National Institutes of Health (NIH), Leidos Biomedical Research, Inc., Frederick National Laboratory for Cancer Research, Frederick, United States; ^6^Center for HIV/AIDS Vaccine Immunology and Immunogen Discovery, The Scripps Research Institute, La Jolla, CA, United States

**Keywords:** HIV-1 Env, soluble trimer, vaccines, antigens, stability, antigenicity

## Abstract

Soluble HIV-1 envelope glycoprotein (Env) trimers are under active investigation as vaccine candidates in relevant pre-clinical models. Like SOSIPs, the cleavage-independent native flexibly linked (NFL) trimers are faithful mimics of the Env spike. Here, we analyzed multiple new designs to explore alternative modifications, informing tertiary interactions, while maintaining NFL trimer homogeneity and integrity. Accordingly, we performed a proline (P) substitution screen in the gp41 heptad repeat 1 region, identifying other trimer-enhancing Ps, including L555P. This P improved trimer integrity compared to I559P in selected properties. Next, we screened 15 structure-guided potential cysteine pairs in gp140 and found that A501C-L663C (“CC2”) forms an inter-protomer disulfide bond that demonstrably increased NFL trimer thermostability. We combined these two approaches with trimer-derived substitutions, coupled with glycine substitutions at helix-to-coil transitions, developed by our group. To increase the exposure of the fusion peptide (FP) N-terminus, we engineered an enterokinase (EK) cleavage site upstream of the FP for controlled post-expression cleavage. In combination, the redesigns resulted in highly stable and homogeneous NFL mimics derived from different clades. Following recombinant EK cleavage, the NFL trimers retained covalent linkage, maintaining a native-like structure while displaying enhanced stability and favorable antigenic features. These trimers also displayed increased exposure of neutralizing epitopes in the FP and gp120/gp41 interface, while retaining other neutralizing epitopes and occluding non-neutralizing elements. This array of Env-structure-guided designs reveals additional interactive regions in the prefusion state of the HIV Env spike, affording the development of novel antigens and immunogens.

## Introduction

Despite many attempts, an effective HIV vaccine remains a daunting scientific challenge. Robust antibody response to HIV Env, the sole target of broadly neutralizing antibodies (bNAbs), is likely required to induce such similar cross-neutralizing Abs following vaccination. However, one big obstacle to develop such a vaccine is to generate a soluble Env that mimics the functional Env trimer present on the surface of the virus. Such native-like trimers should ideally preferentially present bNAb epitopes but shield non-neutralizing epitopes to present to B cells as immunogens *in vivo*.

After decades of development, advances in soluble HIV-1 Env trimer design to permit the generation of a diverse array of native-like trimers (1, 2). Development of the soluble SOSIP.664 trimers provided initial proof-of-principle (3) as these trimers assume a prefusion native-like conformation (4–7). The SOSIPs are proteolytically cleaved by cellular furins to gp120 and gp41 subunits, covalently linked by an engineered intra-protomer disulfide bond A501C-T605C (SOS). These trimers also contain a I559P substitution in the gp41 heptad repeat 1 (HR1) region to generate well-ordered oligomers and, as well, require expression of exogenous furin for conformational integrity (3, 8–17). In the past several years, we developed an improved native-like trimer design, generating well-ordered soluble Env mimics that are completely cleavage-independent and termed native flexibly linked (NFL) trimers. This design uses a flexible linker (two copies of Gly4-Ser, “G_4_S”) to replace the natural cleavage site (18). The linker between the pre-C-terminus of gp120 (residue 507) and N-terminus of gp41 (residue 512) allows the uncleaved trimers to achieve a native-like conformation without the need of furin for precursor processing. The original NFL trimer design contains the I559P mutation as well (18) to disfavor the post-fusion state (3). Both the original SOSIP and NFL designs do not form a high percentage of well-ordered trimers in all Env contexts. For instance, the original NFL design is relatively inefficient in generating high yields of trimers derived from clade C strains, such as 16055 (19).

To improve NFL design, we incorporated residues from BG505 [called trimer-derived (TD) residues] into the 16055 NFLs, improving the propensity to form native-like trimers (19) and, as well, the elicitation of tier 2 clade C neutralizing antibodies (20, 21). Further improvements on the TD design by targeted glycine substitutions at helix-to-coil transitions to disfavor the post-fusion state (TD CC+), significantly improve trimer homogeneity, yield, stability, and antigenicity, resulting in the first high-resolution clade C Env crystal structure (22).

Here, we analyzed three alternative strategies to inform biophysical relationships in the prefusion state while maintaining well-ordered, homogenous, and stable NFL trimers. First, we performed a proline substitution screen across the gp41 HR1 region identifying a new proline substitution (L555P) that improves the generation of stable trimers in some contexts. Second, using structure-guided design, we screened 15 cysteine pairs at different positions, identifying a new cysteine pair A501C-L663C (“CC2”) that forms a stabilizing inter-protomer disulfide bond that is compatible with most TD CC+ substitutions. Third, because the NFL trimers are cleavage-independent (uncleaved), there is limited exposure of the N-terminus of the gp41 fusion peptide (FP) that is recently implicated as broadly neutralizing determinant of the bNAb, VRC34 (23). To restore the exposure of the FP N-terminus, we engineered an EK cleavage site upstream of the FP for controlled post-expression cleavage, greatly enhancing VRC34 recognition. These new designs provide insights regarding critical elements and interactions in the prefusion Env soluble trimer, adding substantially to the plethora of native-like immunogens available for hypothesis-driven, but empirical, immunogenicity determination.

## Materials and Methods

### Design of New NFL Trimer Constructs

**Figure 1:**
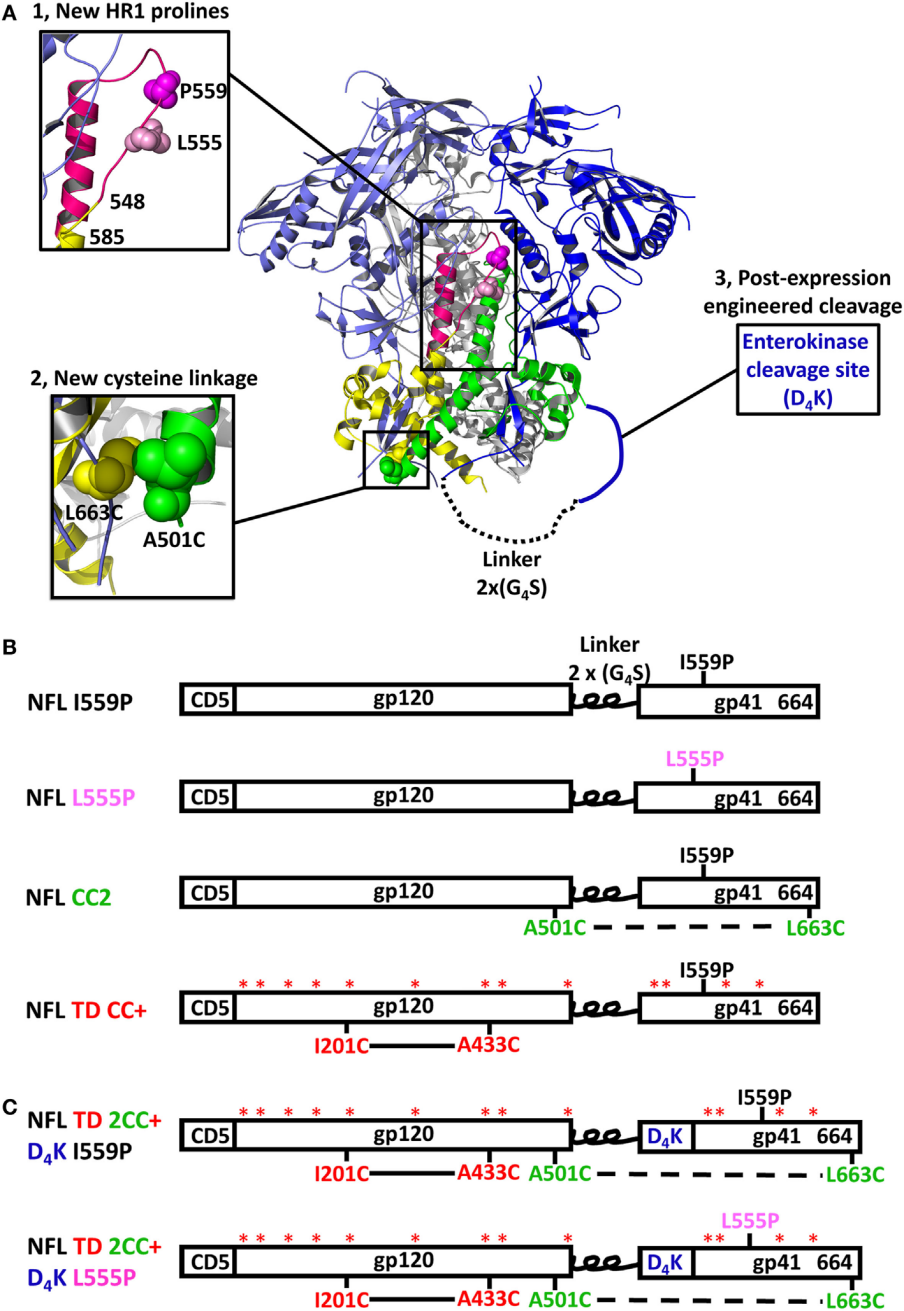
Design and schematic representation of HIV-1 Env NFL trimers. **(A)** Schematic depiction of the approaches used to redesign the NFL trimers using the available BG505 SOSIP.664 structure (PDB: 5CEZ). The trimer is shown in the ribbon representation with an inset of a closer view to illustrate selected approaches of NFL trimer redesign. The gp120 and gp41 in the first gp140 protomer are shown in light blue and yellow, in the second protomer in blue and green, and in the third protomer in gray. Proline substitutions screened in the gp41 heptad repeat 1 region (aa 548–585) are shown in the hot pink ribbon; the first protomer is shown in a close-up view (upper left). L555P is indicated with light pink spheres and I559P by the magenta spheres. The A501C-L663C cysteine pair forming a putative inter-protomer disulfide bond between the first and second protomers is shown in yellow and green spheres in the lower-left, close-up view. The engineered enterokinase (EK) cleavage site is shown as a solid blue line (upstream of the fusion peptide) to allow controlled post-expression cleavage. **(B)** Linear schematic diagram of the NFL with I559P or L555P, A501C-L663C (CC2), or TD CC+ substitutions. **(C)** Linear schematic diagram of the NFL indicating favorable modifications and the engineered EK cleavage site.

The BG505 NFL and 16055 NFL Env sequences (18) (16055 accession numbers EF117268 and BG505 DQ208458) were used as parental templates to generate gp140 trimer mutants. The parental NFL contains a proline substitution at residue 559 (I559P) to facilitate trimer formation (16). For the proline screening, a panel of 36 residues spanning the NFL HR1 region (from residue 548 to 585) was individually substituted by prolines. For the disulfide bond linkage screening, a panel of 15 cysteine pairs was generated. To increase the exposure of epitopes in the FP, we engineered an EK cleavage site (amino acids DDDDK, namely D_4_K) upstream of the FP for controlled post-expression cleavage. Finally, we built promising proline (555P), inter-protomer cysteine linkage (C501–C663), and enterokinase cleavage site into our recently reported NFL TD CC+ trimer constructs (22), namely NFL TD 2CC+ D_4_K (schematic representing the NFL trimer design is shown in Figure 1A). Substitutions in the Env-derived NFL glycoproteins were introduced *via* site-directed mutagenesis PCR (Agilent Technologies) into expression plasmids and confirmed by sequencing (Genewiz). The final constructs are shown as schematic representations in Figures 1B,C.

### Expression and Purification of Soluble Proteins

The constructs expressing the16055 and BG505 NFL trimeric Env were transiently transfected into suspension 293F cells as previously described (18, 19, 22). Env trimer-containing cell culture supernatants were harvested 4 days post transfection and purified by lectin-affinity chromatography (*Galanthus nivalis*, Vector Labs) followed by size exclusion chromatography (SEC) on a Superdex 200 16/60 or Superdex 200 10/300 GL (GE Healthcare). In most cases, the trimer peak was subjected to negative selection (NS) by the non-neutralizing mAbs GE136 or F105 to remove disordered trimers. The flow-through from the GE136 or F105 column, containing the well-ordered trimers, was resolved by a second SEC.

### Immunoprecipitation

Immunoprecipitation (IP) of the expressed Env variants was done as described previously (18) with minor modifications. In brief, ~1 ml of cell culture supernatant was incubated with 5 µg of selected Abs (2G12, VRC01, VRC06, PGT145, PGT151, and F105) at 4°C overnight with rocking. 25 µl of fast-flow protein-A sepharose beads (GE healthcare) were added to each tube and incubated for 1 h at room temperature. Protein-A sepharose beads with the Ab-Env complex was pelleted and washed two times with 1.5 ml of cold PBS with 500 mM NaCl, pH 7.4, and resuspended in 50 µl of 1 × SDS-PAGE loading dye with a reducing agent. The samples were boiled for 10 min, spun to pellet the beads, and the supernatants were loaded onto 4–12% SDS-PAGE gradient gel and run at 200V for 30 min. The gels were stained with 1% tangerine orange dye for 30 min and visualized in the BioRad gel instrument using UV.

### Post-Expression Cleavage of NFL TD 2CC+ D_4_K Trimers by Recombinant Enterokinase (rEK)

Purified NFL TD 2CC+ D_4_K trimers containing engineered EK cleavage site were used for rEK (Novagen) digestion following the manufacturer’s instruction. Briefly, 2 mg of NFL TD 2CC+ D_4_K trimers were cleaved by 50 U rEK at 37°C for 30 h in a buffer containing 20 mM Tris–HCl pH 7.4, 50 mM NaCl, and 2 mM CaCl_2_. The cleavage efficiency was determined by running trimers on SDS-PAGE under reducing and non-reducing conditions (with or without DTT).

### Differential Scanning Calorimetry (DSC)

The thermal transition points (T_m_) of 16055 NFL and BG505 NFL variants were determined by DSC using a MicroCal VP capillary instrument (Malvern) as described previously (18, 22).

### Binding Analyses by ELISA and Biolayer Interferometry (BLI)

ELISA and BLI analyses were performed as previously described (18, 24, 25). Briefly, ELISA plates coated with 2 µg/ml anti-His mAb were used to capture NFL trimers (2 µg/ml) followed by primary mAbs (five-fold serially diluted, starting from 10 µg/ml) and a peroxidase-conjugated goat anti-human secondary Ab (1:10,000). Plates were developed using 3,3′,5,5-tetramethylbenzidine chromagen solution. The data were plotted in GraphPad Prism version 7.

The BLI analyses were carried out on an Octet Red instrument (ForteBio) with IgGs immobilized on anti-human IgG Fc capture sensors (ForteBio). The NFL trimers were assessed as free analytes in solution (PBS pH 7.4). The analytes started from 200 nM and then twofold serially diluted to a final concentration of 12.5 nM. Association and dissociation times were 2 and 2 min or 3 and 3 min, respectively. Data were analyzed using the ForteBio analysis software version 7.1 (ForteBio) and the kinetic parameters were calculated using a global fit 1:1 model.

### Electron Microscopy Data Collection and Processing

The purified trimers were analyzed by negative stain electron microscopy (NS-EM). A 3 µl aliquot containing ~0.01 mg/mL of the sample was applied for 15 s onto a carbon-coated 400 Cu-mesh grid that had been glowing discharged at 30 mA for 30 s, then negatively stained with 0.7% uranyl formate for 45 s. Data were collected using a FEI T20 electron microscope operating at 200 kV, with an electron dose of ~45 e^−^/Å^2^ and a magnification of 80,000× that resulted in a pixel size of 2.74Å at the specimen plane. Images were acquired with an Eagle 2k × 2k CCD camera (FEI) using a nominal defocus of 1,000 nm and the SerialEM software (26). Particles were selected from the micrographs, extracted, and a reference-free 2D class averages were obtained using RELION 2.1.0 (27).

### N-Glycan Profiling of Env Variants With the HR1 P Substitutions

The five BG505 HR1 proline mutants were expressed in 293F cells and purified by the same methods as described above. The overall N-linked glycosylation profiles of these Env variants were analyzed by hydrophilic interaction liquid chromatography-ultraperformance liquid chromatography (HILIC-UPLC) (28). In brief, 10 µg of each of the Envs were resolved by SDS-PAGE under non-reducing and reducing conditions, and the Coomassie blue stained bands corresponding to gp140 were excised and washed five times, alternatively with acetonitrile and water. The total N-linked glycans were enzymatically released by treatment with PNGase F and the released glcyans were washed extensively in water and finally dried in SpeedVac concentrator as described earlier. The released glycans were fluorescently labeled with 2-aminobenzoic acid (2-AA) and resolved on a Acquity BEH Amide column (2.1 mm × 10 mm, 1.7 μm particle size) (Waters) by HILIC-UPLC method as described in detail elsewhere. The raw data were analyzed by Empower 3 software. The relevant peak-areas of different N-linked oligomannose before and after Endo-H digestion were integrated and normalized to calculate the percentage abundance of oligomannose-type glycans in all the Envs.

## Results

### Multiple gp41 HR1 Proline Substitutions Improve Ordered Soluble Env NFL Trimer Formation

The original NFL trimer design contains the I559P mutation (18) to disfavor the post-fusion state (3). However, both the original SOSIP and NFL designs do not form a high percentage of well-ordered trimers in most Env contexts. Here, we introduced single-site proline (P) substitutions in HR1 (residues 548–585) to identify other positions in Env that might efficiently form well-ordered trimers. This screen had added interest to probe the plasticity of Env and the NFL platform in terms of accepting other P substitution in HR1 that were compatible with the pre-fusion state. To begin, we initiated a proline substitution screen in the BG505 context and studied their effects on NFL trimer formation by immunoprecipitation analysis (IP) (18). In total, we interrogated 36 residues in HR1 (Figure 1A) and found that at least five substitutions (S553P, N554P, L555P, Q562P, and Q563P) displayed favorable features based upon trimer-specific bNAb recognition compared to that of the non-neutralizing mAb, F105, in the oligomeric mixture (Figure S1A in Supplementary Material). Among the five substitutions, L555P resulted in ordered trimer production with slightly higher percentage of trimers in a closed native-like conformation (Figures S1B–D in Supplementary Material). These same P substitutions were not efficient in the BG505 SOS 501C/605C context to generate homogeneous trimers, resulting in a broad peak by SEC with no resolution of aggregates, trimers, and dimers/monomers (data not shown). Thermostability analysis by DSC of the five trimer variants revealed that the BG505 NFL L555P trimer was slightly more stable than BG505 NFL I559P trimer, displaying a 1°C increase in T_m_ (Figure S1E in Supplementary Material; Table 1). BG505 NFL L555P trimers exhibited a similar antigenic profile compared to BG505 NFL I559P (Figure S1F in Supplementary Material; Tables 2 and 3). The glycosylation profiles of the five NFL trimer variants were similar as that of BG505 SOSIP.664, but with a higher percentage of oligomannose glycoforms (69.1–78.4%) (Figure S2 in Supplementary Material). The high-density of unprocessed oligomannose glycans in the gp120 subunit of the trimer is consistent with a native-like, closed conformation of these trimers (29, 30), limiting N-glycan enzymatic modifications to more complex glycans.

**Table 1:** Biophysical characterization of stabilized trimers from 16055, BG505, and JRFL isolates.

New substitutions added to parental NFL	Yield (mg/L)	Morphology (NS-EM)	Thermostability (DSC)	
		Native-like (%)	T_m_ (°C)^a^	ΔT_m_ (°C)^b^
**BG505**				

BG505 NFL I559P parental	2.6	97	66.3	–
BG505 NFL S553P	1.8	89	66.3	0.0
BG505 NFL N554P	2.0	90	66.3	0.0
BG505 NFL L555P	2.8	99	67.3	1.0
BG505 NFL Q562P	1.7	46	66.4	0.1
BG505 NFL Q563P	2.0	40	66.7	0.4
BG505 NFL CC2	1.2	94	70.4	4.1
BG505 NFL TD CC+	2.0	98	77.0	10.7
BG505 NFL TD 2CC+ D_4_K L555P	2.5	93	80.9	14.6
BG505 NFL TD 2CC+ D_4_K L555P w/ rEK	2.1	98	81.6	15.3
BG505 NFL TD 2CC+ D_4_K I559P	2.7	98	80.4	14.1
BG505 NFL TD 2CC+ D_4_K I559P w/ rEK	2.2	98	81.0	14.7

**16055**				

16055 NFL I559P parental^c^	0.2	90	58.8	–
16055 NFL L555P	1.8	98	57.7	−1.1
16055 NFL Q562P	1.9	94	57.5	−1.3
16055 NFL Q563P	2.0	95	59.3	0.5
16055 NFL CC2	1.0	98	65.4	6.6
16055 NFL TD CC+^c^	2.5	98	77.0	18.2
16055 NFL TD 2CC+ D_4_K L555P	2.6	97	80.2	21.4
16055 NFL TD 2CC+ D_4_K L555P w/ rEK	2.1	94	82.8	24.0
16055 NFL TD 2CC+ D_4_K I559P	2.8	98	80.1	21.3
16055 NFL TD 2CC+ D_4_K I559P w/ rEK	2.4	98	82.6	23.8

**JRFL**				

JRFL NFL I559P parental^c^	1.0	15	54.3	–
JRFL NFL CC2	1.8	94	59.3	5.0

*The yields of purified trimers after negative selection are listed, together with the percentages of closed native-like conformation determined by NS-EM. The 2D class averages from NS-EM of the trimers are shown in related figures and Supplementary figures*.

*^a^The T_m_ values were obtained by DSC analyses*.

*^b^ΔT_m_ represents the change of melting temperature of the modified trimer compared to parental NFL I559P trimer*.

*^c^Data shown here are adapted from previously published studies (19, 22)*.

**Table 2:** Antigenic characterization of stabilized trimers from 16055, BG505, and JRFL isolates.

New substitutions added to parental NFL	Broadly neutralizing antibodies (bNAbs)								Non-neutralizing antibodies			
	
	2G12	PGDM1400	PGT145	PG16	PGT151	VRC01	VRC34	PGT128	447-52D	F105	GE136	17b
**16055**												

16055 NFL I559P parental	2.339	+/ND	+/ND	+/ND	>10	0.055	0.692	+/ND	>10	>10	0.913	0.438
16055 NFL L555P	0.051	0.019	0.014	0.121	0.048	0.178	+/ND	0.056	>10	>10	+/ND	+/ND
16055 NFL Q563P	0.066	0.016	0.013	0.102	0.047	0.294	>10	0.068	>10	>10	+/ND	+/ND
16055 NFL CC2	0.056	0.018	0.013	0.089	>10	0.021	0.434	0.135	>10	>10	>10	>10
16055 NFL TD CC+	0.046	0.021	0.022	0.142	>10	0.049	>10	0.044	>10	>10	>10	>10
16055 NFL TD 2CC+ D_4_K I559P	0.043	0.025	0.015	0.123	>10	0.059	>10	0.055	>10	>10	>10	>10
16055 NFL TD 2CC+ D_4_K I559P w/ rEK	0.105	0.030	0.019	0.190	0.041	0.208	0.034	0.080	>10	>10	>10	>10
16055 NFL TD 2CC+ D_4_K L555P	0.038	0.021	0.014	0.115	>10	0.067	>10	0.050	>10	>10	>10	>10
16055 NFL TD 2CC+ D_4_K L555P w/ rEK	0.110	0.025	0.015	0.122	0.056	0.178	0.032	0.071	>10	>10	>10	>10

**BG505**												

BG505 NFL I559P parental	0.029	0.039	0.016	0.144	0.062	0.050	0.464	0.039	>10	+/ND	+/ND	+/ND
BG505 NFL L555P	0.022	0.020	0.018	0.101	0.058	0.070	0.017	0.021	>10	+/ND	+/ND	+/ND
BG505 NFL CC2	0.025	0.018	0.011	0.143	0.059	0.059	0.457	0.035	>10	>10	>10	+/ND
BG505 NFL TD CC+	0.022	0.034	0.011	0.185	0.062	0.036	>10	0.035	>10	>10	>10	>10
BG505 NFL TD 2CC+ D_4_K I559P	0.024	0.038	0.013	0.206	0.065	0.042	0.072	0.036	>10	>10	>10	>10
BG505 NFL TD 2CC+ D_4_K I559P w/ rEK	0.031	0.038	0.016	0.336	0.091	0.066	0.037	0.036	>10	>10	>10	>10
BG505 NFL TD 2CC+ D_4_K L555P	0.027	0.042	0.014	0.215	0.073	0.051	2.427	0.041	>10	>10	>10	>10
BG505 NFL TD 2CC+ D_4_K L555P w/ rEK	0.021	0.040	0.015	0.307	0.085	0.069	0.035	0.037	>10	>10	>10	>10

**JRFL**												

JRFL NFL CC2	0.022	0.201	0.012	0.216	0.139	0.032	1.009	0.040	6.117	>10	>10	>10

*Binding of bNAbs and non-NAbs was determined using a His-tag capture ELISA. Half-maximal binding concentrations (EC_50_, in µg/ml) are shown in the table. The values are representative of at least two independent experiments. The ELISA curves from one representative experiment are shown in Supplementary figures. +/ND, indicates that the binding reactivity was positive but was not high enough to reliably determine an EC_50_ value*.

**Table 3:** Kinetic parameters of the NFL trimers with trimer-preferring V2-apex and cleavage-sensitive bNAbs.

New substitutions added to parental NFL		PGDM1400	PGT145	PG16	PGT151	VRC34
**16055**						

16055 NFL I559P parental^a^	K_D_ (nM)	**78**	**116**	**34**	NT	NT
	K_on_ (1/Ms) × 10^4^	2.9	3.2	3.1	NT	NT
	K_off_ (1/s) × 10^−3^	2.3	3.7	1.0	NT	NT

16055 NFL L555P	K_D_ (nM)	**23**	**33**	**34**	**25**	NT
	K_on_ (1/Ms) × 10^4^	20	31	26	29	NT
	K_off_ (1/s) × 10^−3^	4.5	10	8.7	7.4	NT

16055 NFL Q562P	K_D_ (nM)	**14**	**29**	**28**	**26**	NT
	K_on_ (1/Ms) × 10^4^	23	30	17	29	NT
	K_off_ (1/s) × 10^−3^	3.1	8.7	4.8	7.6	NT

16055 NFL Q563P	K_D_ (nM)	**13**	**16**	**17**	**26**	NT
	K_on_ (1/Ms) × 10^4^	30	32	20	30	NT
	K_off_ (1/s) × 10^−3^	3.8	5.3	3.3	7.8	NT

16055 NFL CC2	K_D_ (nM)	**14**	**16**	**15**	**21**	**63**
	K_on_ (1/Ms) × 10^4^	11	24	6.1	25	4.3
	K_off_ (1/s) × 10^−3^	1.6	4.0	0.9	5.3	2.7

16055 NFL TD CC+^a^	K_D_ (nM)	**15**	**18**	**17**	**15**	**60**
	K_on_ (1/Ms) × 10^4^	7.6	13	4.5	27	8.9
	K_off_ (1/s) × 10^−3^	1.2	2.3	0.8	4.2	5.3

16055 NFL TD 2CC+ D_4_K L555P	K_D_ (nM)	**13**	**16**	**16**	**13**	**75**
	K_on_ (1/Ms) × 10^4^	12	22	4.3	45	5.0
	K_off_ (1/s) × 10^−3^	1.6	3.5	0.7	5.5	3.8

16055 NFL TD 2CC+ D_4_K I559P	K_D_ (nM)	**21**	**25**	**23**	**8.7**	**92**
	K_on_ (1/Ms) × 10^4^	9.8	22	4.3	79	4.1
	K_off_ (1/s) × 10^−3^	2.1	5.6	1.0	6.9	3.8

**BG505**						

BG505 NFL I559P parental	K_D_ (nM)	**11**	**14**	**15**	**5.5**	NT
	K_on_ (1/Ms) × 10^4^	17	15	20	39	NT
	K_off_ (1/s) × 10^−3^	1.8	2.3	3.0	2.2	NT

BG505 NFL L553P	K_D_ (nM)	**11**	**12**	**7.2**	**3.3**	NT
	K_on_ (1/Ms) × 10^4^	18	19	24	53	NT
	K_off_ (1/s) × 10^−3^	1.9	2.0	1.7	1.7	NT

BG505 NFL N554P	K_D_ (nM)	**5.5**	**16**	**14**	**1.4**	NT
	K_on_ (1/Ms) × 10^4^	20	13	16	41	NT
	K_off_ (1/s) × 10^−3^	1.1	2.1	2.2	0.6	NT

BG505 NFL L555P	K_D_ (nM)	**11**	**13**	**13**	**1.4**	NT
	K_on_ (1/Ms) × 10^4^	17	15	5	48	NT
	K_off_ (1/s) × 10^−3^	1.9	1.9	2.0	0.7	NT

BG505 NFL Q562P	K_D_ (nM)	**13**	**12**	**15**	**1.9**	NT
	K_on_ (1/Ms) × 10^4^	11	17	19	47	NT
	K_off_ (1/s) × 10^−3^	1.5	2.1	2.7	0.9	NT

BG505 NFL Q563P	K_D_ (nM)	**11**	**14**	**12**	**1.7**	NT
	K_on_ (1/Ms) × 10^4^	16	16	21	48	NT
	K_off_ (1/s) × 10^−3^	1.8	2.2	2.5	0.8	NT

BG505 NFL CC2	K_D_ (nM)	**24**	**21**	**43**	**1.9**	**1.3**
	K_on_ (1/Ms) × 10^4^	7.6	16	7.8	29	2.7
	K_off_ (1/s) × 10^−3^	1.8	3.4	3.3	0.6	0.04

BG505 NFL TD CC+^a^	K_D_ (nM)	**20**	**22**	**45**	**3.5**	**47**
	K_on_ (1/Ms) × 10^4^	6.5	9.4	7.1	19	2.5
	K_off_ (1/s) × 10^−3^	1.3	2.1	3.2	0.7	1.2

BG505 NFL TD 2CC+ D_4_K L555P	K_D_ (nM)	**23**	**19**	**70**	**3.7**	**44**
	K_on_ (1/Ms) × 10^4^	8.8	17	8.1	37	4.4
	K_off_ (1/s) × 10^−3^	2.0	3.1	5.7	1.4	2.0

BG505 NFL TD 2CC+ D_4_K I559P	K_D_ (nM)	**18**	**16**	**59**	**2.3**	**18**
	K_on_ (1/Ms) × 10^4^	8.8	17	8.1	34	8.8
	K_off_ (1/s) × 10^−3^	1.6	2.7	4.8	0.8	1.6

*The above table displays the affinity parameters measured by biolayer interferometry (BLI) of the NFL trimers to the trimer-preferring V2-apex and the cleavage-sensitive bNAbs. KD values (nM) are highlighted in bold. NT, not tested*.

*^a^Data shown here are adapted from previously published studies (19, 22)*.

**Figure 2:**
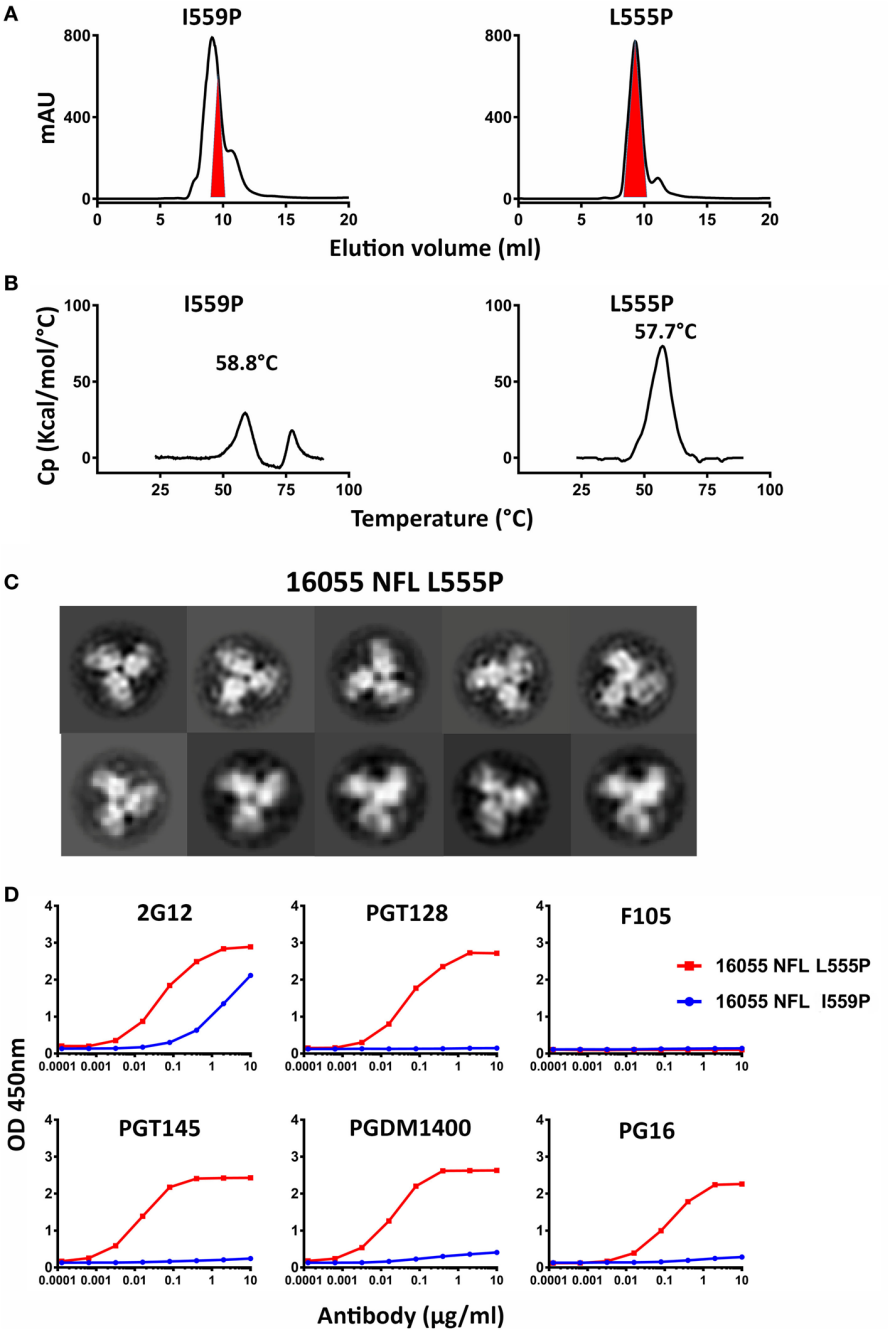
Biochemical, biophysical, and antigenic characterization of 16055 NFL trimers possessing the L555P substitution. **(A)** Comparison of the size exclusion chromatography profiles of the 16055 NFL trimers possessing the I559P and L555P substitutions following lectin-affinity purification. The shaded red area indicates the native-like trimer fractions. The yields are summarized in Table 1. **(B)** Differential scanning calorimetry measurements of 16055 NFL trimers possessing either I559P or L555P. The data for 16055 NFL I559P are adapted as previously reported (19). The T_m_ values are shown on top of the peaks and are summarized in Table 1. **(C)** 2D class averages from negative stain electron microscopy (NS-EM) of 16055 NFL L555P trimers purified by negative selection using GE136. **(D)** ELISA binding of selected mAbs to the NFL trimers and the half-maximal binding concentrations (EC_50_, in µg/ml) are summarized in Table 2.

Because the HR1 region is relatively conserved among Env from different clades, we determined whether the P substitutions identified in the BG505 NFL context could be transferred to the clade C 16055 NFL, which is inefficient in its original I559P design in terms of yielding a high percentage of native-like trimers (19). All five proline substitutions (S553P, N554P, L555P, Q562P, and Q563P) were compatible within the 16055 NFL backbone (Figure S3A in Supplementary Material), displaying a homogenous trimer peak by SEC. In contrast, the 16055 NFL I559P SEC peak contained only small fractions of trimers (Figure 2A; Figure S3B in Supplementary Material), as previously reported (19). The trimer peak on SEC was slightly shifted “to the right” for 16055 NFL L555P design, consistent with improved trimer formation and yield (Table 1). However, some trimer heterogeneity remained, so we used GE136-affinity negative selection to remove non-native trimers and other Env conformers. Following negative selection, the yield of the 16055 NFL L555P trimers were increased compared to the original I559P trimers (Table 1). DSC analysis revealed that the L555P substitution generated more homogenous 16055 NFL trimers, with comparable thermostability, compared to the I559P change (Figure 2B; Figure S3C in Supplementary Material). NS-EM 2D average analysis showed that >94% of the trimers were in closed native-like conformation (Figure 2C; Figure S3D in Supplementary Material; Table 1). Antigenicity analysis by BLI and ELISA showed that L555P substitution improved the antigenic profile of 16055 NFL trimers, revealing increased recognition by the trimer-specific V2-apex-directed bNAbs (PGT145, PGDM1400, and PG16) and V3-targeting bNAb (PGT128), but little-to-no detectable binding by the non-NAbs (F105, GE136, 17b, and 447-52D) (Figure 2D; Figures S3E,F in Supplementary Material; Tables 2 and 3).

Taken together, these data show that the L555P substitution is comparable to, or improved, relative to the original I559P substitution regarding to form well-ordered, homogenous, and stable NFL trimers.

### An Inter-Protomer Disulfide Bond Improves the Stability and Antigenicity of the Soluble NFL Trimers

To reduce the flexibility and increase the stability of the first generation of NFL I559P trimers, we sought to identify additional internal disulfide pairs to stabilize NFL trimers. Accordingly, we performed cysteine disulfide predictions based on the distance between Cα atoms (<5.7Å), taking into consideration a low probability of disrupting secondary structure. Guided by the existing SOSIP and NFL structures, we down-selected 15 potential cysteine pairs, predicted to be within the 5.7Å side-chain distance, in the most likely rotamers (listed in Table S1 in Supplementary Material). We first assessed these potential new disulfide linkages in the original JRFL NFL I559P context. From the IP results, we identified a new cysteine pair (A501C-L663C, designated here as “CC2”) showing favorable recognition by PGT145, VRC06, and PGT151, and low-level recognition by F105 (data not shown).

**Figure 3:**
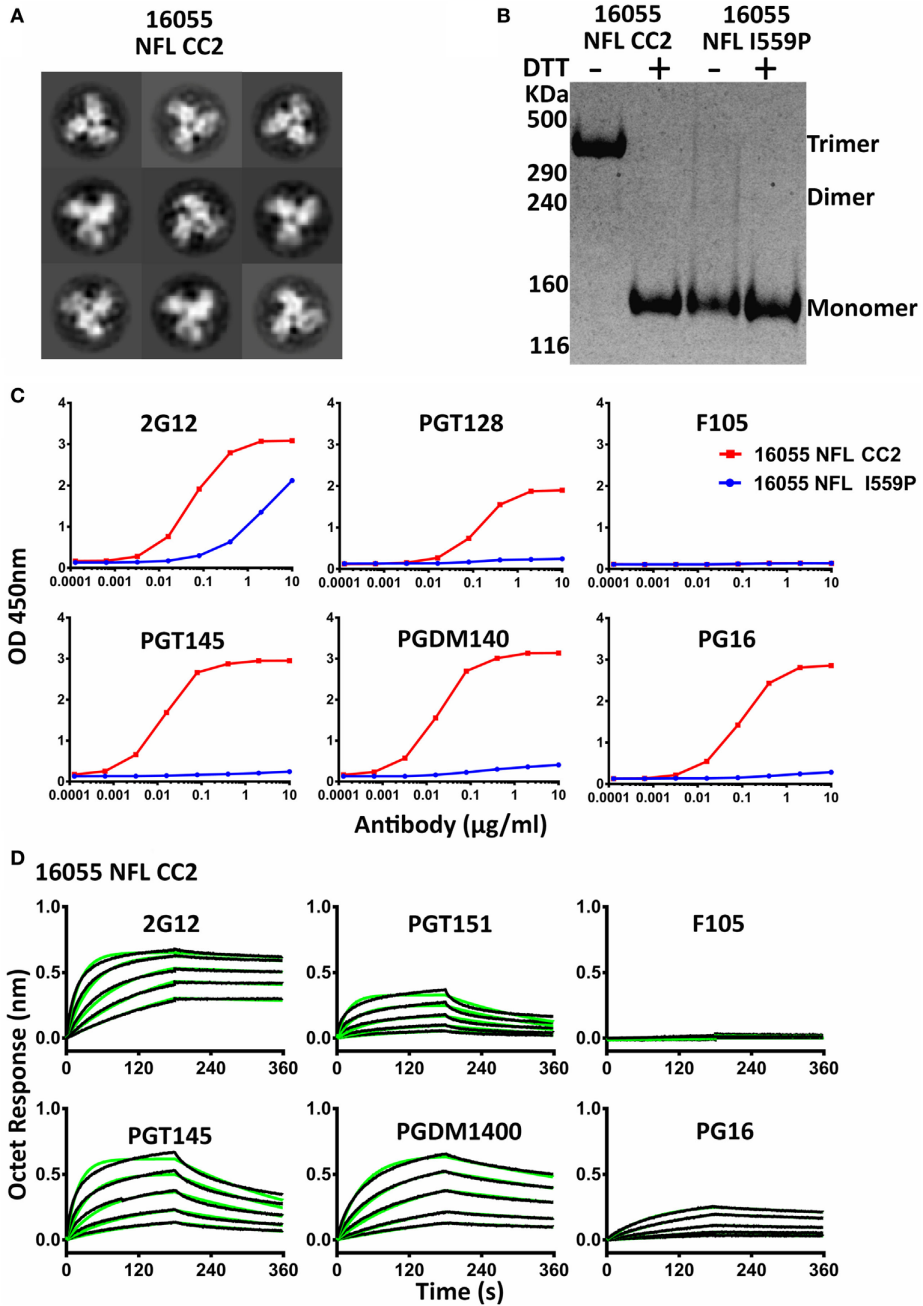
Biochemical, biophysical, and antigenic properties of 16055 NFL CC2 trimers. **(A)** 2D class averages from negative stain electron microscopy of 16055 NFL CC2 trimers. **(B)** Disulfide bond formation was determined by SDS-PAGE under reducing and non-reducing conditions, respectively. Under reducing conditions, all proteins displayed a gp140 species. Under non-reducing conditions, the CC2 proteins displayed a trimeric gp140 species, migrating more slowly in the gel. **(C)** ELISA binding of selected mAbs to the 16055 NFL CC2 trimers and the EC_50_ values are summarized in Table 2. **(D)** BLI measurements for 16055 NFL CC2 trimers with selected mAbs. The fitting curves are shown in green and the kinetic parameters are summarized in Table 3.

We analyzed the CC2 cysteine pair in NFL Envs derived from different clades, JRFL (clade B), 16055 (clade C), and BG505 (clade A). All three NFL trimers containing the engineered CC2 cysteine substitutions form well-ordered trimers, displaying a single sharp trimer peak by SEC (Figure S4A in Supplementary Material). The purified trimers were resolved on Blue-native PAGE (BN-PAGE), revealing a migration pattern consistent with predominantly trimeric Env. A low level of apparent dimer forms was detected for the new NFL CC2 design (Figure S4B in Supplementary Material). Homogeneous trimer formation was confirmed by NS-EM 2D class average as the CC2 trimers were highly ordered following negative staining and the EM analysis (Figure 3A; Figure S4D in Supplementary Material; Table 1). To better confirm efficient 501–663 disulfide bond formation, we performed the gel analysis without and with reduction. Under reducing and non-reducing conditions, by SDS-PAGE analysis, the NFL I559P (without CC2) trimer proteins migrated as gp140 monomer. However, the NFL CC2 proteins migrated as trimer under non-reducing conditions, whereas under reducing conditions they migrated as gp140 monomer (Figure 3B; Figure S4C in Supplementary Material). These results were consistent with inter-protomer disulfide bond formation, covalently linking adjacent protomers to form well-ordered trimers.

Differential scanning calorimetry analysis revealed that the new inter-protomer disulfide bond increased the thermostability of the NFL trimers, for example, the presence of the 501–663 CC2 in the NFL backbone increased the T_m_ by 6.6°C for 16055 trimers, 4.1°C for BG505, and 5.0°C for JRFL trimers (Figure S4E in Supplementary Material; Table 1). The antigenic profile of the NFL CC2 trimers analyzed by BLI and ELISA showed that CC2 in 16055 NFL improved trimer recognition by the trimer-specific bNAbs (PGT145, PGDM1400, and PG16) and V3-targeting bNAb (PGT128) (Figures 3C,D; Tables 2 and 3). In BG505 NFL, the improvement of the CC2 substitutions regarding antigenicity was evident, but to a lesser extent (Figures S4F,G in Supplementary Material).

Taken together, these data indicate that the new cysteine pair A501C-L663C (CC2) formed inter-protomer disulfide bonds, increasing the thermostability and antigenicity of NFL trimers derived from different clades.

### Combinatorial Approaches Improve NFL Trimer Biophysical Properties and Antigenicity

As described above, the L555P substitution and the new CC2 inter-protomer disulfide bond improved the NFL trimer design, separately. In addition, we recently reported that TD residue substitutions, glycine substitution at helix-to-coil transitions, as well as targeted reduction of the inherent Env metastability facilitate the high-yield production of cross-clade stable soluble NFL TD CC+ trimers. Therefore, we combined these design strategies in the 16055 and BG505 NFL Env context, to generate NFL TD 2CC+ trimers. We assessed whether these combined designs were cross-compatible to yield improved, well-ordered trimers. In addition, since our NFL trimers are uncleaved, there is limited exposure of the N-terminus of the gp41 FP recognized by the bNAb, VRC34, and variable accessibility to the cleavage-sensitive bNAb, PGT151 depending upon the strain context (Figures S3E and S4F,G in Supplementary Material). To increase the exposure and accessibility to the cleavage-sensitive bNAbs, especially the gp41 FP N-terminus, we engineered an enterokinase (EK) cleavage site upstream of the FP. This modification would allow us to control post-expression cleavage of gp140, potentially exposing the VRC34-FP-directed binding site (outlined in Figure 1). The FP was recently reported as a vulnerable target of the VRC34 and ACS202 bNAbs (23, 31). The resulting trimers were designated as NFL TD 2CC+ D_4_K. For head-to-head comparison, we generated two versions of NFL TD 2CC+ D_4_K trimers in 16055 and BG505 backbone, one containing the original I559P substitution and another possessing the identified L555P substitution.

**Figure 4:**
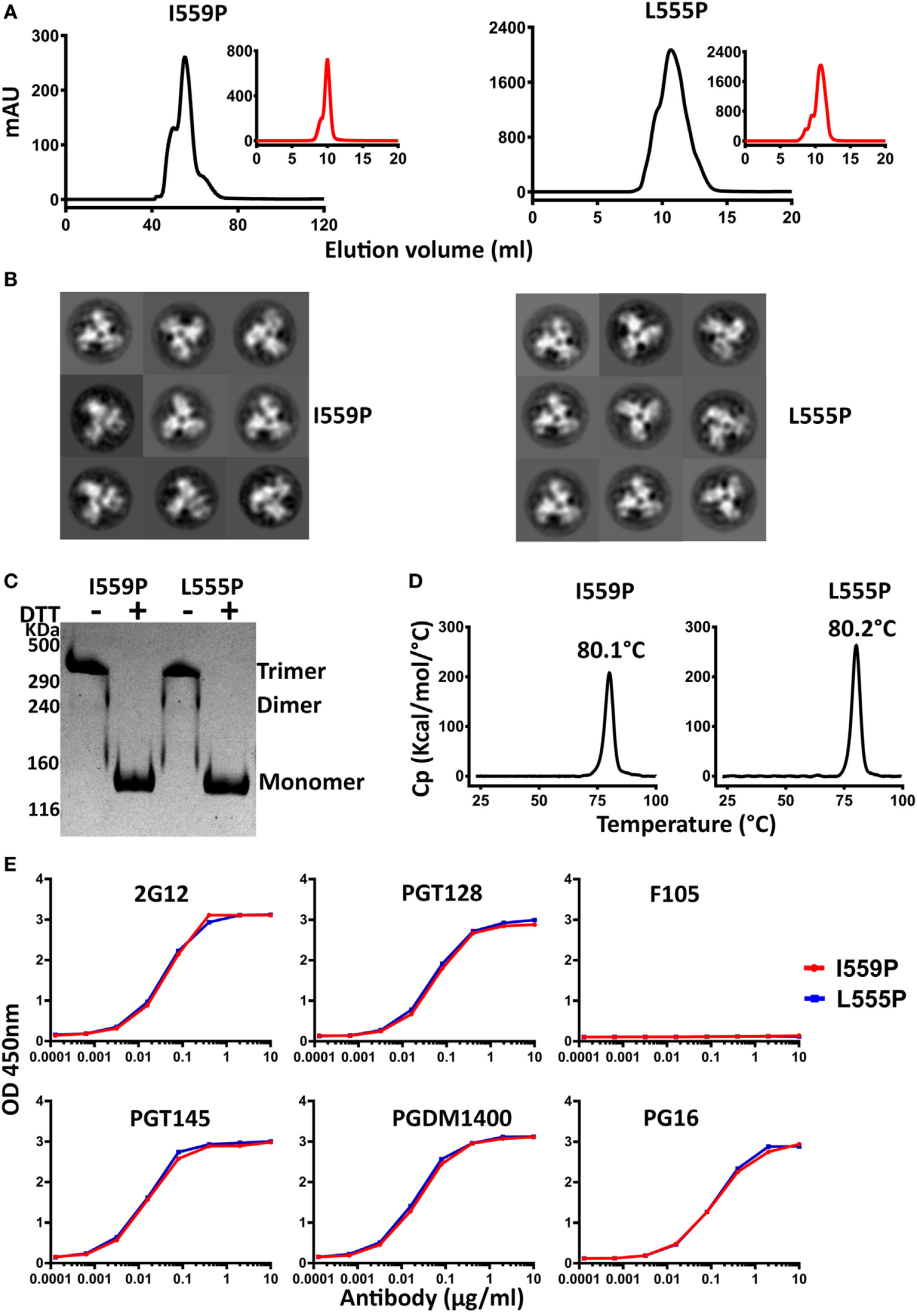
Biochemical, biophysical, and antigenic characterization of 16055 NFL TD 2CC+ D_4_K trimers possessing the I559P and L555P substitutions. **(A)** Size exclusion chromatography (SEC) profiles of 16055 NFL TD 2CC+ D_4_K I559P and L555P trimers following lectin-affinity purification. SEC profiles after GE136 negative selection are shown in the insets. **(B)** Comparison of 2D class averages from negative stain electron microscopy of 16055 NFL TD 2CC+ D_4_K I559P and L555P trimers. **(C)** Disulfide bond formation was determined by SDS-PAGE under reducing and non-reducing conditions. **(D)** Differential scanning calorimetry measurements of 16055 NFL TD 2CC+ D_4_K I559P and L555P trimers. The T_m_ values are shown above the peaks, and summarized in Table 1. **(E)** Comparison of ELISA binding properties of selected mAbs to 16055 NFL TD 2CC+ D_4_K I559P and L555P trimers. The EC_50_ are summarized in Table 2.

The 16055 and BG505 NFL TD 2CC+ D_4_K L555P and I559P variants were purified by lectin-affinity chromatography, followed by SEC. The SEC trimer peak of 16055 NFL TD 2CC+ D_4_K I559P was much sharper than that of 16055 NFL TD 2CC+ D_4_K L555P, indicating the I559P substitution is more compatible with these modifications compared to L555P (Figure 4A). As expected, the SEC profile revealed a single sharp trimer peak following NS. Similar SEC profiles were observed for BG505 NFL TD 2CC+ D_4_K L555P and I559P (Figure S6A in Supplementary Material). Regardless of the P substitution used, the combinatorial design in 16055 dramatically increased the yield of well-ordered trimers by over 13-fold compared to original 16055 NFL I559P (Table 1). Trimer formation was confirmed by BN-PAGE (Figures S5A and S6C in Supplementary Material). In addition, NS-EM analysis revealed that >97% trimers in closed native-like conformation (Figure 4B; Figure S6B in Supplementary Material; Table 1). Under non-reducing conditions, the NFL TD 2CC+ D_4_K proteins migrated as trimer on the SDS-PAGE, whereas under reducing conditions these proteins migrated as a gp140 monomer, consistent with the formation of inter-protomer disulfide bonds following the CC2 substitutions (Figure 4C; Figure S6D in Supplementary Material).

Differential scanning calorimetry analysis revealed that the T_ms_ of these trimers were over 80°C for both NFL TD 2CC+ D_4_K L555P and I559P, displaying an increase of over 21°C for 16055 NFL TD 2CC+ D_4_K and over 14°Cfor BG505 NFL TD 2CC+ D_4_K, relative to the “first generation” NFL I559P trimers (Figure 4D; Figure S6E in Supplementary Material). There was no significant difference of thermostability between NFL TD 2CC+ D_4_K L555P and I559P, but there was over a 3°C gain for NFL TD 2CC+ D_4_K compared to their corresponding NFL TD CC+ trimers, indicating the addition of CC2 increased thermal stability (Table 1). The NFL TD 2CC+ D_4_K L555P and I559P trimers were highly stable in solution, displaying no significant degradation at 37°C for 30 h indicated by gel analysis (data not shown). We used the previously described panel of bNAbs and non-neutralizing Abs to assess the antigenicity of 16055 NFL TD 2CC+ D_4_K L555P and I559P trimers by ELISA and BLI, revealing that both trimer variants were recognized comparably by the trimer-specific bNAbs with no detectable recognition by the non-neutralizing Abs tested (Figure 4E; Figure S5 in Supplementary Material; Tables 2 and 3). Comparable antigenicity profiles were similarly detected for BG505 NFL TD 2CC+ D_4_K L555P and I559P trimers (Figures S6F,G in Supplementary Material), consistent with trimer integrity.

Taken together, these analyses demonstrated that the combination of L555P, CC2, TD CC+, and engineered post-expression cleavage site, preserve the pre-fusion state of the NFL trimers with improved trimer formation, biophysical properties, and antigenicity.

### Post-Expression Cleavage of NFL TD 2CC+ D_4_K Trimers Increases the Exposure of Cleavage-Sensitive Epitopes

**Figure 5:**
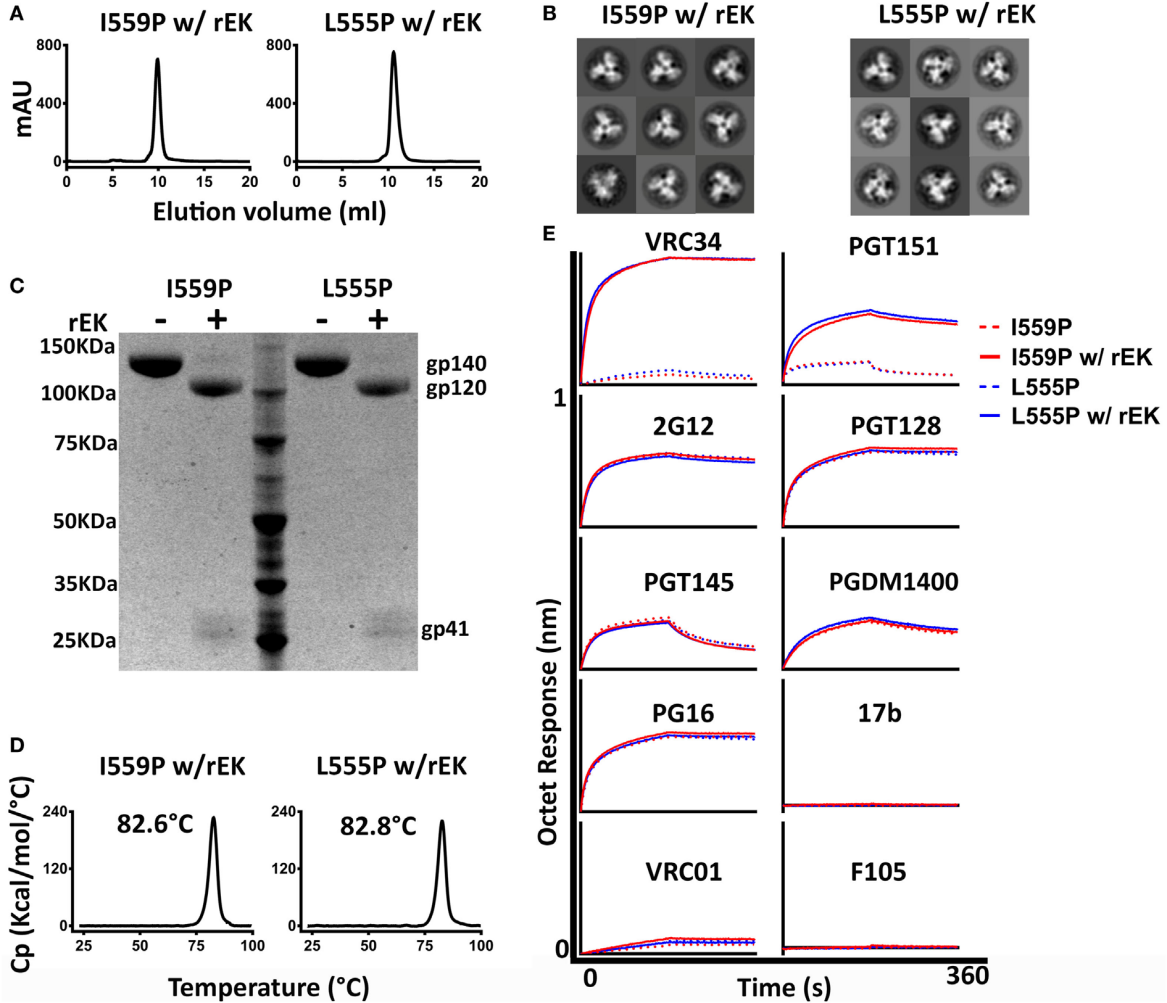
Biochemical, biophysical, and antigenic characterization of 16055 NFL TD 2CC+ D_4_K I559P and L555P trimers after recombinant enterokinase (rEK) cleavage. **(A)** Size exclusion chromatography profiles of 16055 NFL TD 2CC+ D_4_K I559P and L555P trimers after rEK cleavage (w/ rEK). **(B)** Comparison of 2D class averages from negative stain electron microscopy of cleaved 16055 NFL TD 2CC+ D_4_K I559P and L555P trimers (w/ rEK). **(C)** Cleavage efficiency was determined by SDS-PAGE under reducing conditions. **(D)** Differential scanning calorimetry measurements of cleaved 16055 NFL TD 2CC+ D_4_K I559P and L555P trimers (w/ rEK). The T_m_ values are shown above the peak and are summarized in Table 1. **(E)** BLI comparison for the interactions of uncleaved and cleaved (w/ rEK) 16055 NFL TD 2CC+ D_4_K trimers with selected mAbs. The kinetic parameters are summarized in Table 3.

Next, we assessed the impact of post-expression cleavage on the highly stable NFL TD 2CC+ D_4_K trimers regarding their structure, biophysical properties, and antigenicity. Following cleavage by rEK, 16055 NFL TD 2CC+ D_4_K L555P and I559P trimers showed single sharp trimer peaks on SEC (Figure 5A) with >94% of the trimers in a closed native-like conformation as resolved by NS-EM (Figure 5B). Similar results were observed for BG505 NFL TD 2CC+ D_4_K L555P and I559P trimers (Figures S7A,B in Supplementary Material). Under native conditions, cleaved 16055 NFL TD 2CC+ D_4_K proteins migrated as trimer on BN-PAGE, similar to their uncleaved counterparts (Figure S5A in Supplementary Material). To test the efficiency of post-expression cleavage, we performed SDS-PAGE analysis. Under reducing conditions, cleaved 16055 NFL TD 2CC+ D_4_K L555P and I559P proteins migrated as two bands, gp120 and gp41, whereas the uncleaved proteins migrated as a single gp140 band (Figure 5C), indicating the trimers were completely cleaved by rEK. Similar results were obtained for BG505 NFL TD 2CC+ D_4_K L555P and I559P trimers (Figure S7C in Supplementary Material).

Following rEK-mediated cleavage, DSC analysis of putative trimers revealed single narrow symmetric thermal transition profiles, indicating that the trimers were homogeneous (Figure 5D; Figure S7D in Supplementary Material). The T_ms_ of the cleaved 16055 NFL TD 2CC+ D_4_K L555P and I559P trimers were 82.8 and 82.6°C, respectively, displaying 2.6 and 2.5°C increases compared to their uncleaved counterparts. The T_ms_ of cleaved BG505 NFL TD 2CC+ D_4_K L555P and I559P trimers were 81.6 and 81.0°C, respectively, with 0.7 and 0.6°C increases over their uncleaved counterparts (Table 1). We used a panel of bNAbs and non-neutralizing Abs to assess the antigenicity changes of 16055 NFL TD 2CC+ D_4_K L555P and I559P trimers after cleavage by ELISA and BLI. Following rEK cleavage, the 16055 NFL TD 2CC+ D_4_K L555P and I559P trimers displayed increased recognition by the cleavage-sensitive bNAbs VRC34 and PGT151, while retaining similar levels of recognition by bNAbs targeting other epitopes (2G12, VRC01, and PGT128) (Figure 5E; Figure S8A in Supplementary Material). In addition, there was no recognition by the non-neutralizing Abs (F105, GE136, 17b, and 447-52D). Similar antigenic profile was observed for BG505 NFL TD 2CC+ D_4_K L555P and I559P trimers after cleavage as well (Figures S7E and S8B in Supplementary Material).

Overall, these data indicate that the CC2 covalently linked the rEK-cleaved trimers to maintain native-like structure with enhanced stability and increased exposure of epitopes in the FP, gp120/gp41 interface.

## Discussion

Here, we describe two NFL redesign strategies, namely proline substitution in HR1 and introduction of an inter-protomer disulfide bond, to generate soluble NFL trimers with improved biophysical properties. By single proline substitution in HR1, we identify several positions that favor the prefusion state of Env, similar to 559P. In terms of well-ordered soluble NFL trimer formation, the L555P substitution is the most comparable to, and in some context, even superior to the I559P substitution. Based on structure-guided cysteine pair analysis, we found that A501C-L663C (CC2) forms an inter-protomer disulfide bond that stabilizes the original uncleaved NFL trimer. We combined these two approaches to generate NFL TD CC+ trimers, containing the CC2 inter-protomer linkage in combination with the intra-protomer 201–433 CC disulfide bond. To restore more efficient recognition by the FP-directed bNAb, VRC34, we substituted an EK cleavage site upstream of the FP for controlled post-expression cleavage. We show that following cleavage, the NFL TD 2CC+ D_4_K trimers were covalently linked by CC2 to maintain native-like structure, displaying enhanced stability and increased exposure of epitopes in the FP and gp120/gp41 interface. Thus, Env-structure-guided trimer redesign results in homogenous cross-clade immunogens with the potential to advance vaccine development, Ab isolation, and Env structural analysis.

The ability of multiple proline substitutions in HR1 to generate varying degrees of well-ordered NFL trimers in the oligomeric population is interesting and consistent with the concept that this region contributes to metastability. In fact, one approach to generate cleavage-independent native-like trimers modifies the FP and portions of HR1 to generate stable, well-ordered UFO trimers (32). Guenaga et al. reported that glycine substitutions at key coil-to-helix HR1 transitions favor trimer formation in the prefusion state (22). Another study shows that the interface between α6 in HR1 and α9, and the inter-subunit β-sheet are critical for trimer stability (33). This is likely related to HIV gp41 metastability in the context of the native spike. Following gp120 engagement with receptor/co-receptor, the spike undergoes conformational changes from a putative high-energy pre-hairpin conformation to a low-energy, stable post-fusion six-helix bundle (6-HB) conformation, mediating viral-to-host cell membrane fusion. Mutations K574R and I535M in HR1 are reported to increased Env stability in different clades (34, 35), and V570D and I573D can destabilize the 6-HB formation (36), favoring the prefusion state on the cell-surface, independent of the I559P substitution. The SOS-defined I559P substitution is an important modification to stabilize soluble trimer mimics in the prefusion conformation for SOSIPs (3, 8–17, 33), single-chain gp140 (37), DS-SOSIP (38, 39), and the NFL trimers (18, 19, 22). Here, the L555P identified in our P screen is comparable to or even better than I559P in some NFL contexts but is not compatible with the BG505 SOS backbone in this regard, indicating additional advantages of the NFL design to accept specific engraftments, perhaps since it lacks the engineered 501–605 disulfide.

Internal engineered disulfide bonds clearly increase trimer protein stability. For example, the respiratory syncytial virus F protein trimers (dSCAV) and influenza virus HA soluble trimers provide proof-of-principle (40). For HIV Env, the well-documented intra-protomer SOS bond covalently linking gp120 residue 501 to gp41 residue 605 impacts favorably on trimer formation (3, 7, 16, 17). A second intra-protomer disulfide bond linking gp120 residues 201–433, stabilizes the bridging sheet in its prefusion state and thereby reducing conformational changes and potential CD4-induced exposure of non-neutralizing Abs epitopes in both NFL and SOSIP trimers (19, 39). Two additional disulfide bonds have been introduced into BG505 SOSIP and generate hyper-stable native-like trimers (BG505 SOSIP.v6) but results in lower trimer yields (41). Here, we identified a new cysteine pair called CC2 (A501C-L663C) that forms an inter-protomer disulfide bond and increases the thermostability of multiple NFLs, augmenting trimer formation while maintaining desired antigenicity. Although the engineered disulfide is efficiently formed, it does generate some “off-pathway” dimers for reasons that are not yet clear.

Size exclusion chromatography analyses of NFL TD 2CC+ D_4_K trimers reveals that I559P is more compatible with the CC2 and TD CC+ modifications than is either the L555P or Q563P substitutions (data not shown), indicating that further optimization is needed to render the L555P more compatible in combination with the CC2 and TD CC+ substitutions. To approach this issue, we performed NS to remove heterogeneous, non-native-like trimers from all NFL TD 2CC+ D_4_K variants. Following the NS step, all trimer variants are homogenous and highly stable (>80°C T_m_), without degradation at 37°C for 30 h. Following post-expression cleavage by rEK, the thermostability of trimers increases as much as 2.6°C, while maintaining a favorable antigenic profile. Uncleaved NFL trimers exhibit isolate-specific effects regarding the exposure of the PGT151 and VRC34 epitopes, and particular stabilizing modification also affect the accessibility of these epitopes in the uncleaved context. However, following post-expression cleavage by rEK, trimer recognitions are greatly increased for VRC34 and PGT151 relative to most uncleaved states, indicating increased exposure of these binding sites. Although rEK post-cleavage adds another processing step for the NFL trimers, this approach does allow such trimers to be cleaved in a controlled manner to expose the N-terminus of the FP for improved presentation of the VRC34 epitope on NFL inter-protomer disulfide-linked immunogens, if so desired.

In sum, by the multi-fold modifications described here, we generated highly stable prefusion, closed, and cleavage-independent Env trimers from multiple strains with favorable antigenic and biochemical properties that are amenable for HIV vaccine development. The design strategies may also be applicable to stabilize fusion proteins derived from other enveloped viruses that undergo conformational changes in transitioning from pre-fusion to post-fusion states as candidate vaccines or for structural analysis.

## Author Contributions

LY, SS, and RTW designed research studies. LY, SS, CC, RW, A-JB, and NV performed experiments and data analysis. LY and RTW wrote the paper. All authors reviewed the results and approved the final version of the manuscript.

## Conflict of Interest Statement

The authors declare that the research was conducted in the absence of any commercial or financial relationships that could be construed as a potential conflict of interest.

## Supplementary Material

Figure S1 Proline substitution screening in BG0505 NFL heptad repeat 1 region. **(A)** Representative IP data of BG505 NFL trimer variants with selected proline substitutions. **(B)** Size exclusion chromatography (SEC) profiles of BG505 NFL trimers with selected proline substitutions. Aggregates in the SEC of 16055 NFL I559P are shown in red dash box. Yields are summarized in Table 1. **(C)** Blue-native PAGE (BN-PAGE) analyses of proteins taken from SEC trimer peaks. **(D)** 2D averages from negative stain electron microscopy of BG505 NFL trimer variants. The percentage of closed native-like and open native-like trimers is shown in red and black, as well as the percentage of non-native trimers in blue. The data are summarized in Table 1. **(E)** Differential scanning calorimetry measurements of BG505 NFL trimer variants. The T_m_ values are shown on top of the peaks, and summarized in Table 1. **(F)** Biolayer interferometry measurements for trimers interaction with selected mAbs. The kinetic parameters are summarized in Table 3.

Figure S2 Glycan profiles of BG505 NFL trimers with selected proline substitutions. The glycan profiles of trimer variants are determined by hydrophilic interaction liquid chromatography-ultraperformance liquid chromatography. The peaks colored in green shades represent the oligomannose (Man_5–9_GlcNAc_2_) and hybrid-type glycans, and peaks colored in pink represent the remaining complex glycans. The areas under the peaks converted into percentage occupancy of glycans are plotted correspondingly in the form of pie-chart and summarized in the table below. The BG505 NFL proline variants have higher percentage of oligomannose (especially Man_8–9_GlcNAC_2_) than the SOSIP.664 counterpart.

Figure S3 Proline substitution screening in 16055 NFL heptad repeat 1 region. **(A)** IP analyses of 16055NFL trimer variants with selected proline substitutions. **(B)** Size exclusion chromatography (SEC) profiles of 16055 NFL trimer variants following lectin-affinity purification. The shaded red area indicates the native-like trimer fractions. **(C)** Differential scanning calorimetry measurements of 16055 NFL trimer variants. The T_m_ values are shown on top of the peaks. **(D)** 2D averages from negative stain electron microscopy of NFL trimer variants purified by negative selection using GE136. **(E)** ELISA binding of selected mAbs to the NFL trimers. The EC_50_ values are summarized in Table 2. **(F)** BLI measurements for trimers interaction with selected mAbs. The kinetic parameters are summarized in Table 3.

Figure S4 Characterization of NFL CC2 trimers from different clades. **(A)** Size exclusion chromatography profiles of JRFL (clade B), BG505 (clade A), and 16055 (clade C) NFL CC2 trimers after lectin-affinity purification, and followed by negative selection (NS). **(B)** Blue-native PAGE analyses of trimers before and after NS. **(C)** Disulfide bond formation was determined by SDS-PAGE under reducing (+DTT) and non-reducing (−DTT) conditions, respectively. **(D)** 2D class averages from negative stain electron microscopy of JRFL, BG505, and 16055 NFL CC2 trimers. **(E)** Differential scanning calorimetry measurements of NFL CC2 trimers. The T_m_ values are shown on top of the peaks. **(F)** ELISA binding of selected mAbs to NFL CC2 trimers. The EC_50_ values are summarized in Table 2. **(G)** BLI measurements for BG505 and 16055 NFL CC2 trimers with selected mAbs. The fitting curves are shown in green color, and the kinetic parameters are summarized in Table 3.

Figure S5 Characterization of 16055 NFL TD 2CC+ D_4_K I559P and L555P trimers. **(A)** Blue-native PAGE analyses of 16055 NFL TD 2CC+ D_4_K trimers without or with recombinant enterokinase (rEK) cleavage. **(B)** Comparison of ELISA binding properties of selected mAbs to 16055 NFL TD 2CC+ D_4_K I559P and L555P trimers without rEK cleavage. The EC_50_ values are summarized in Table 2. **(C)** BLI measurements of 16055 NFL TD 2CC+ D_4_K I559P and L555P trimers without rEK cleavage. The fitting curves are shown in blue color, and the kinetic parameters are summarized in Table 3.

Figure S6 Characterization of BG505 NFL TD 2CC+ D_4_K I559P and L555P trimers. **(A)** size exclusion chromatography (SEC) profiles of BG505 NFL TD 2CC+ D_4_K I559P and L555P trimers following lectin-affinity purification. SEC profiles after F105 negative selection are shown in the insets. **(B)** 2D averages from negative stain electron microscopy of BG505 NFL TD 2CC+ D_4_K I559P and L555P trimers. **(C)** Blue-native PAGE analyses of BG505 NFL TD 2CC+ D_4_K I559P and L555P trimers with or without recombinant enterokinase (rEK) cleavage. **(D)** Disulfide bond formation was determined by SDS-PAGE under reducing and non-reducing conditions, respectively. **(E)** Differential scanning calorimetryr measurements of BG505 NFL TD 2CC+ D_4_K I559P and L555P trimers without rEK cleavage. **(F)** Comparison of ELISA binding properties of selected mAbs to BG505 NFL TD 2CC+ D_4_K I559P and L555P trimers without rEK cleavage. The EC_50_ values are summarized in Table 2. **(G)** BLI measurements of BG505 NFL TD 2CC+ D_4_K I559P and L555P trimers without cleavage. The fitting curves are shown in blue color and the kinetic parameters are summarized in Table 3.

Figure S7 Characterization of BG505 NFL TD 2CC+ D_4_K I559P and L555P trimers after recombinant enterokinase (rEK) cleavage. **(A)** Size exclusion chromatography profiles of BG505 NFL TD 2CC+ D_4_K I559P and L555P trimers after rEK cleavage (w/ rEK). **(B)** Comparison of 2D averages from negative stain electron microscopy of BG505 NFL TD 2CC+ D_4_K I559P and L555P trimers after rEK cleavage. **(C)** Cleavage efficiency was determined by SDS-PAGE under reducing conditions. **(D)** Differential scanning calorimetry measurements of BG505 NFL TD 2CC+ D_4_K I559P and L555P trimers after rEK cleavage. **(E)** BLI measurements for BG505 NFL TD 2CC+ D_4_K I559P and L555P trimers interaction with selected mAbs before and after rEK cleavage. The kinetic parameters are summarized in Table 3.

Figure S8 Comparison of ELISA binding reactivities between NFL TD 2CC+ D_4_K I559 and L555P trimers after recombinant enterokinase (rEK) cleavage. **(A)** Comparison of ELISA binding properties of selected mAbs to 16055 NFL TD 2CC+ D_4_K I559 and L555P trimers after rEK cleavage. **(B)** Comparison of ELISA binding properties of selected mAbs to BG505 NFL TD 2CC+ D_4_K I559 and L555P trimers after rEK cleavage. The EC_50_ values are summarized in Table 2.
