# Supplementary figures and images for: Structure-Guided Redesign Improves NFL HIV Env Trimer Integrity and Identifies an Inter-Protomer Disulfide Permitting Post-Expression Cleavage
Source: Front Immunol. 2018 Jul 17;9:1631. doi: 10.3389/fimmu.2018.01631 (PMC6056610; doi:10.3389/fimmu.2018.01631)

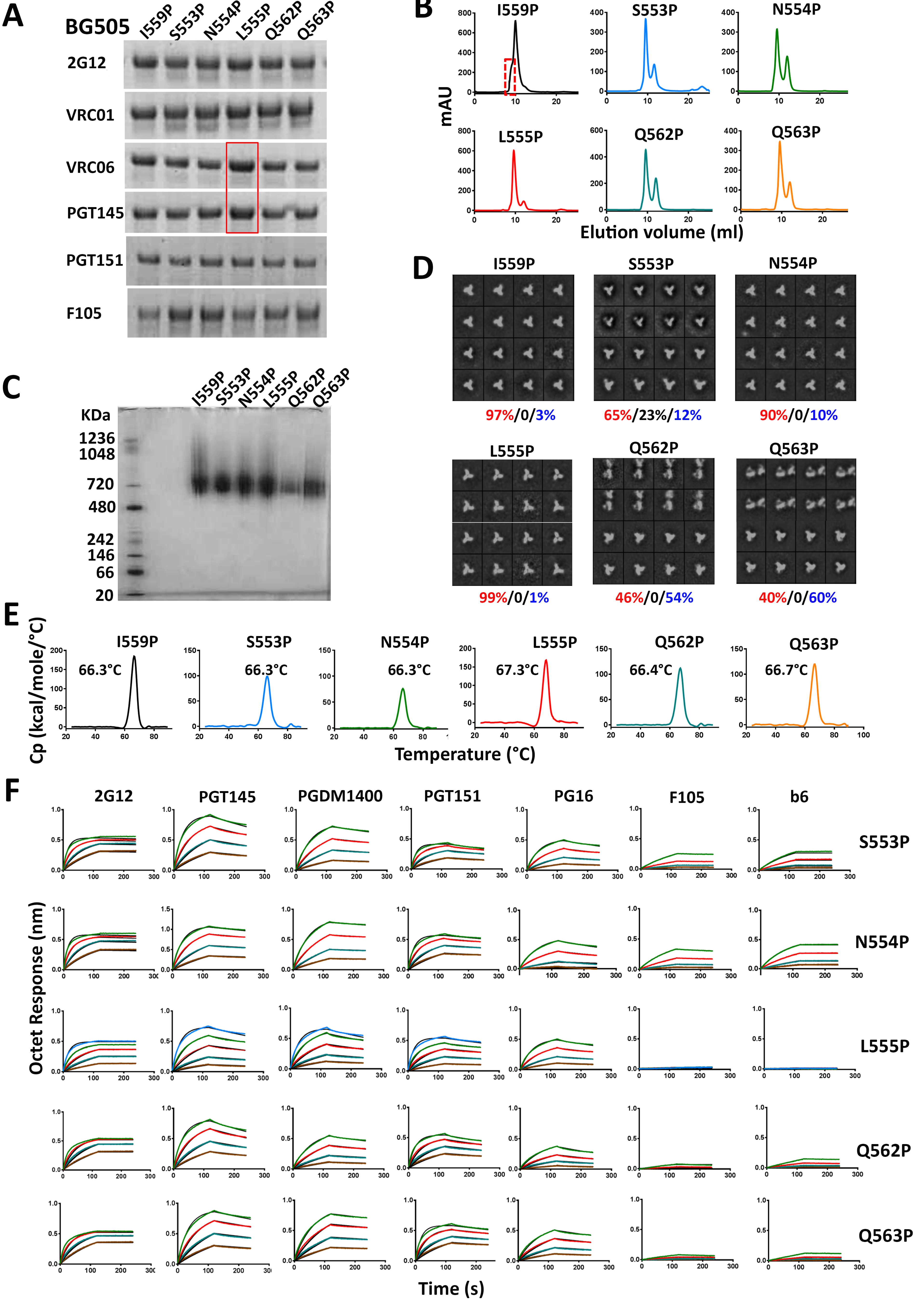

Supplement: Figure S1 — Proline substitution screening in BG0505 NFL heptad repeat 1 region. (A) Representative IP data of BG505 NFL trimer variants with selected proline substitutions. (B) Size exclusion chromatography (SEC) profiles of BG505 NFL trimers with selected proline substitutions. Aggregates in the SEC of 16055 NFL I559P are shown in red dash box. Yields are summarized in Table 1. (C) Blue-native PAGE (BN-PAGE) analyses of proteins taken from SEC trimer peaks. (D) 2D averages from negative stain electron microscopy of BG505 NFL trimer variants. The percentage of closed native-like and open native-like trimers is shown in red and black, as well as the percentage of non-native trimers in blue. The data are summarized in Table 1. (E) Differential scanning calorimetry measurements of BG505 NFL trimer variants. The Tm values are shown on top of the peaks, and summarized in Table 1. (F) Biolayer interferometry measurements for trimers interaction with selected mAbs. The kinetic parameters are summarized in Table 3. [file Image_1.tif]

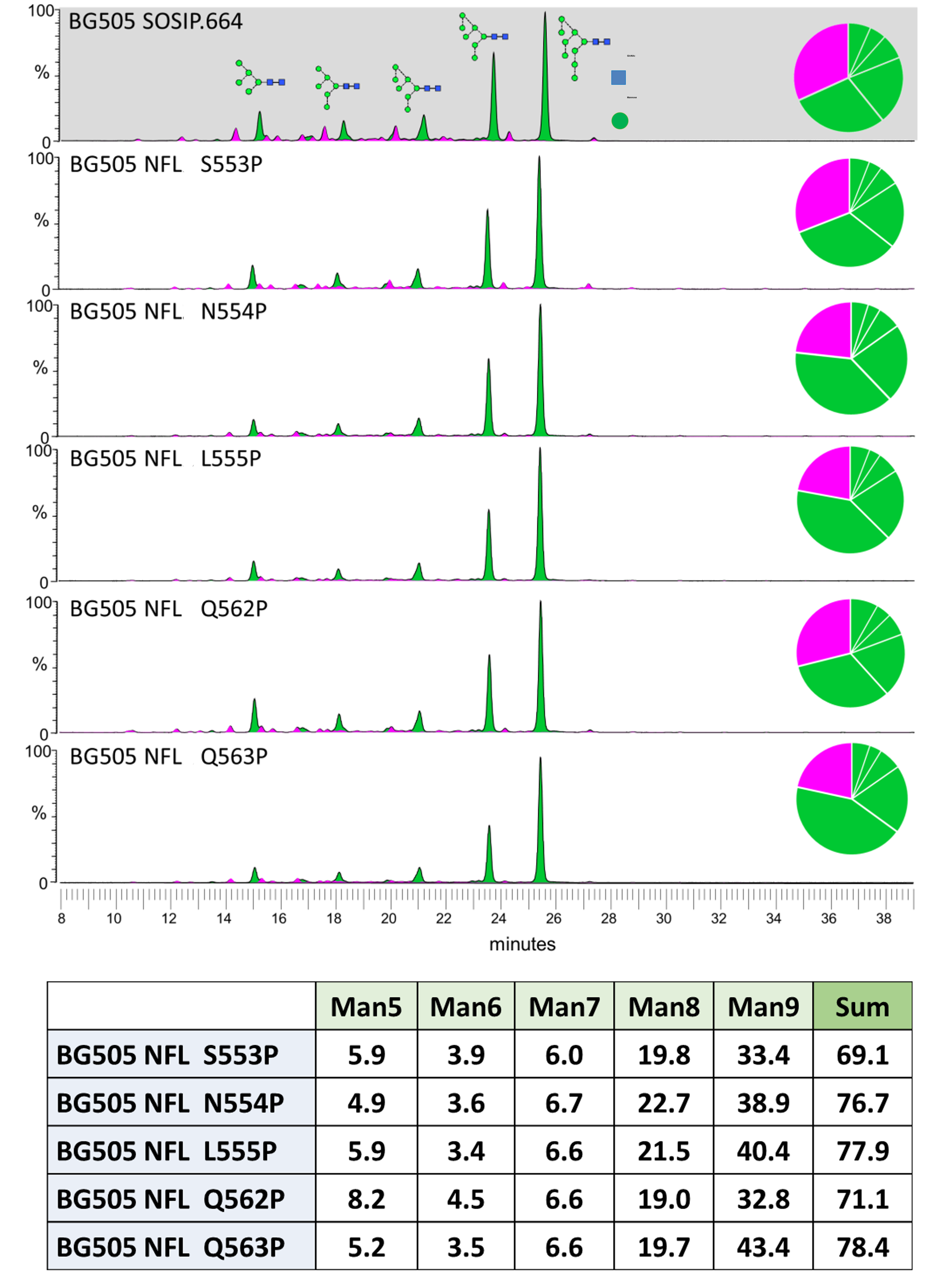

Supplement: Figure S2 — Glycan profiles of BG505 NFL trimers with selected proline substitutions. The glycan profiles of trimer variants are determined by hydrophilic interaction liquid chromatography-ultraperformance liquid chromatography. The peaks colored in green shades represent the oligomannose (Man5–9GlcNAc2) and hybrid-type glycans, and peaks colored in pink represent the remaining complex glycans. The areas under the peaks converted into percentage occupancy of glycans are plotted correspondingly in the form of pie-chart and summarized in the table below. The BG505 NFL proline variants have higher percentage of oligomannose (especially Man8–9GlcNAC2) than the SOSIP.664 counterpart. [file Image_2.tif]

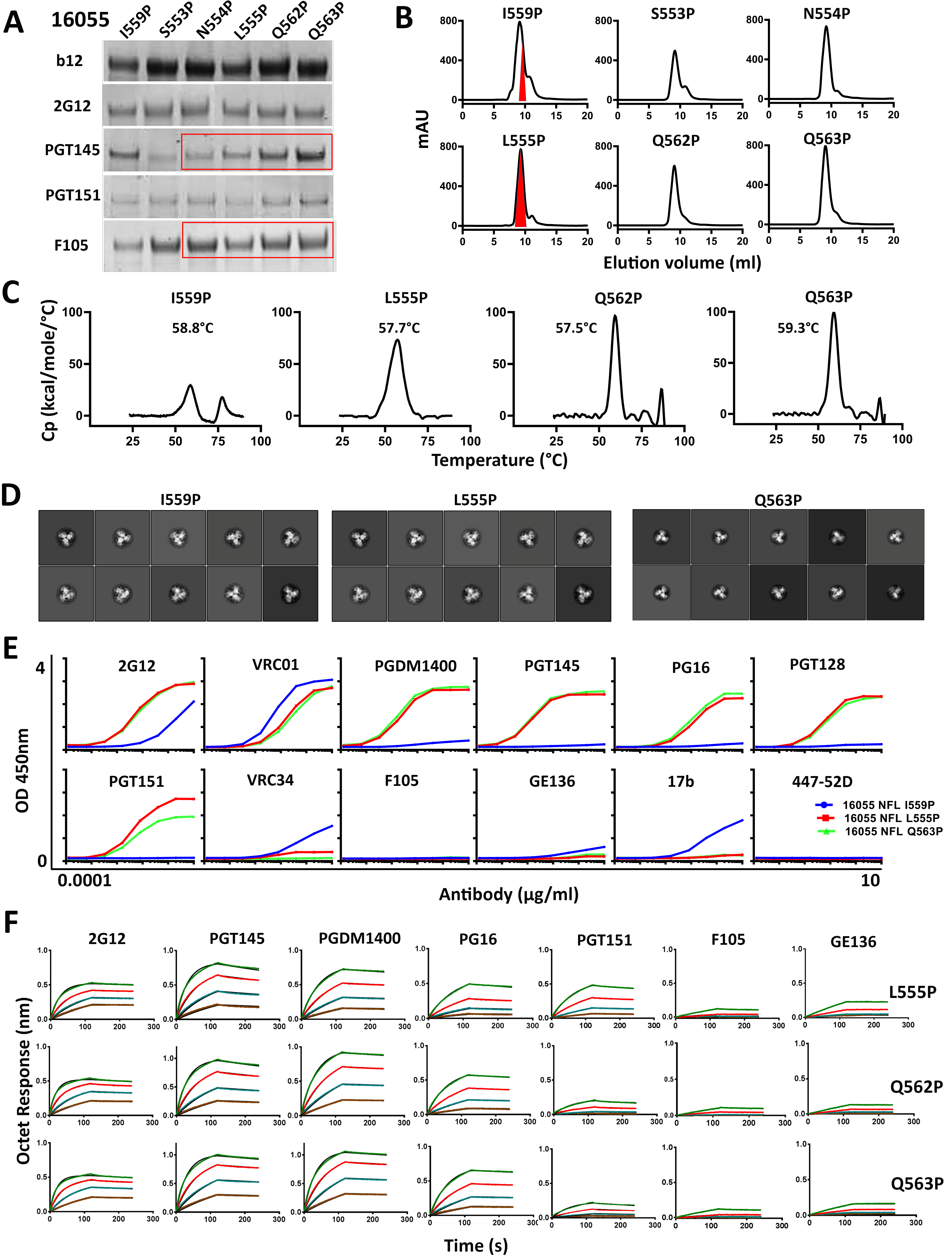

Supplement: Figure S3 — Proline substitution screening in 16055 NFL heptad repeat 1 region. (A) IP analyses of 16055NFL trimer variants with selected proline substitutions. (B) Size exclusion chromatography (SEC) profiles of 16055 NFL trimer variants following lectin-affinity purification. The shaded red area indicates the native-like trimer fractions. (C) Differential scanning calorimetry measurements of 16055 NFL trimer variants. The Tm values are shown on top of the peaks. (D) 2D averages from negative stain electron microscopy of NFL trimer variants purified by negative selection using GE136. (E) ELISA binding of selected mAbs to the NFL trimers. The EC50 values are summarized in Table 2. (F) BLI measurements for trimers interaction with selected mAbs. The kinetic parameters are summarized in Table 3. [file Image_3.tif]

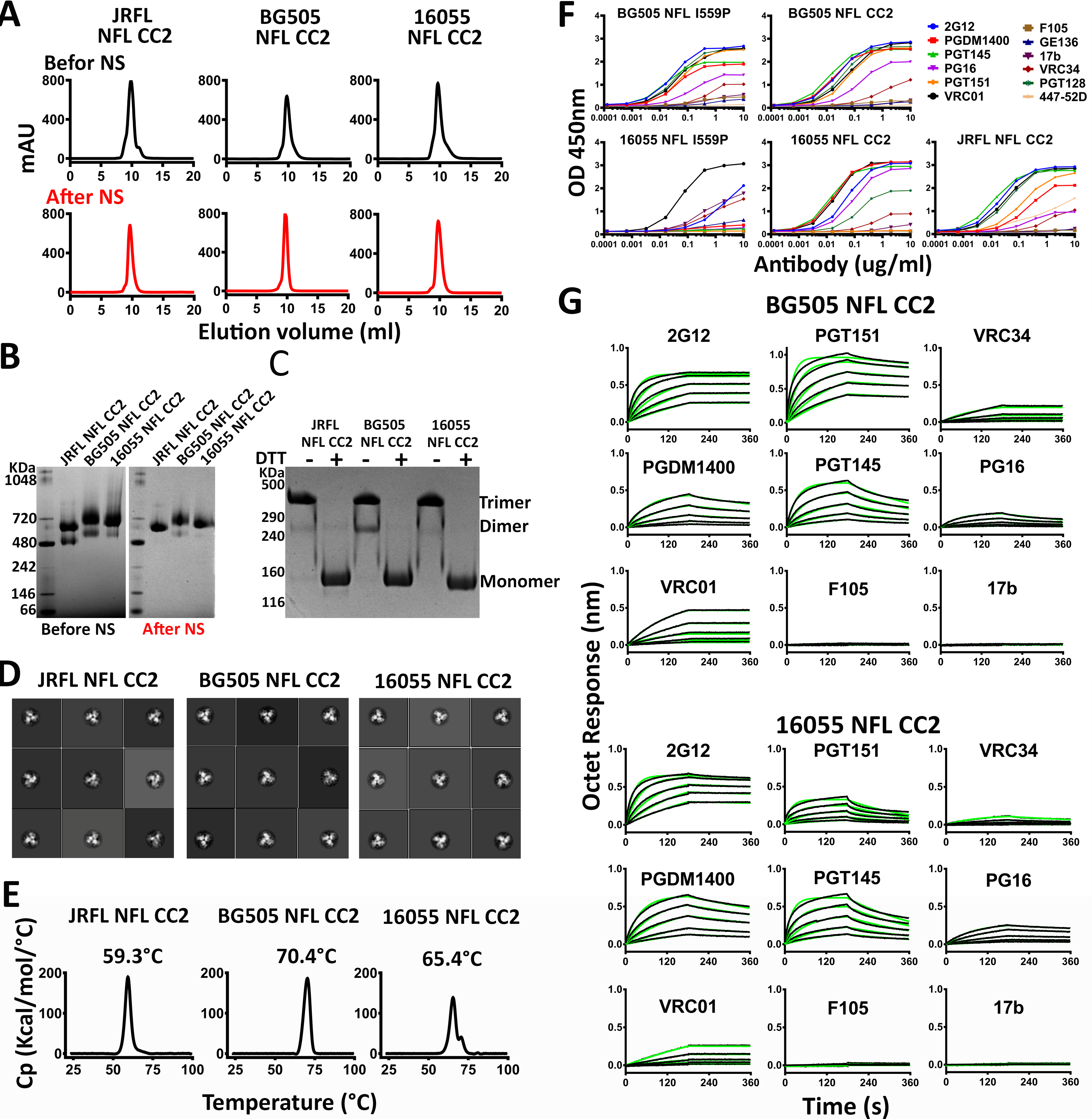

Supplement: Figure S4 — Characterization of NFL CC2 trimers from different clades. (A) Size exclusion chromatography profiles of JRFL (clade B), BG505 (clade A), and 16055 (clade C) NFL CC2 trimers after lectin-affinity purification, and followed by negative selection (NS). (B) Blue-native PAGE analyses of trimers before and after NS. (C) Disulfide bond formation was determined by SDS-PAGE under reducing (+DTT) and non-reducing (−DTT) conditions, respectively. (D) 2D class averages from negative stain electron microscopy of JRFL, BG505, and 16055 NFL CC2 trimers. (E) Differential scanning calorimetry measurements of NFL CC2 trimers. The Tm values are shown on top of the peaks. (F) ELISA binding of selected mAbs to NFL CC2 trimers. The EC50 values are summarized in Table 2. (G) BLI measurements for BG505 and 16055 NFL CC2 trimers with selected mAbs. The fitting curves are shown in green color, and the kinetic parameters are summarized in Table 3. [file Image_4.tif]

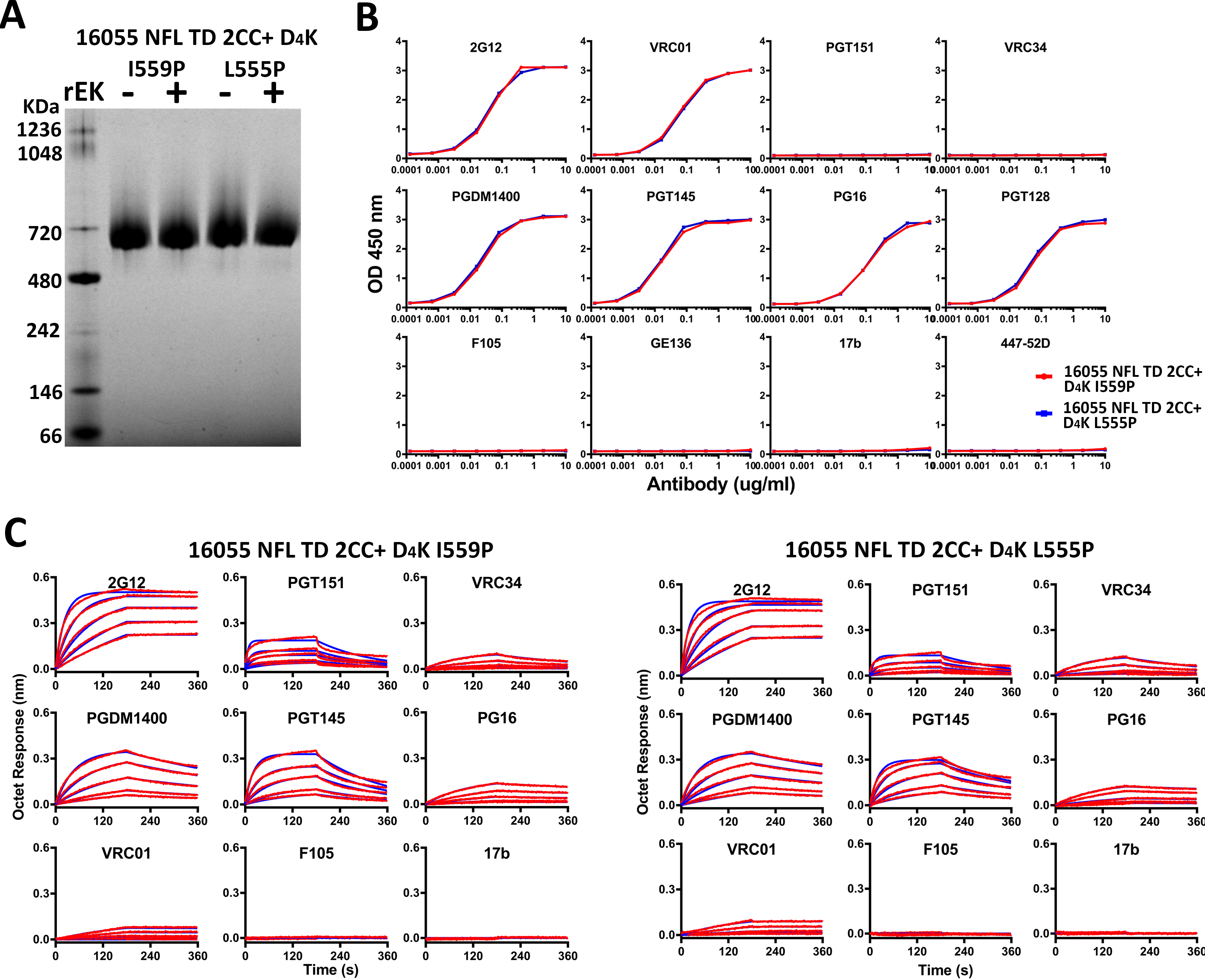

Supplement: Figure S5 — Characterization of 16055 NFL TD 2CC+ D4K I559P and L555P trimers. (A) Blue-native PAGE analyses of 16055 NFL TD 2CC+ D4K trimers without or with recombinant enterokinase (rEK) cleavage. (B) Comparison of ELISA binding properties of selected mAbs to 16055 NFL TD 2CC+ D4K I559P and L555P trimers without rEK cleavage. The EC50 values are summarized in Table 2. (C) BLI measurements of 16055 NFL TD 2CC+ D4K I559P and L555P trimers without rEK cleavage. The fitting curves are shown in blue color, and the kinetic parameters are summarized in Table 3. [file Image_5.tif]

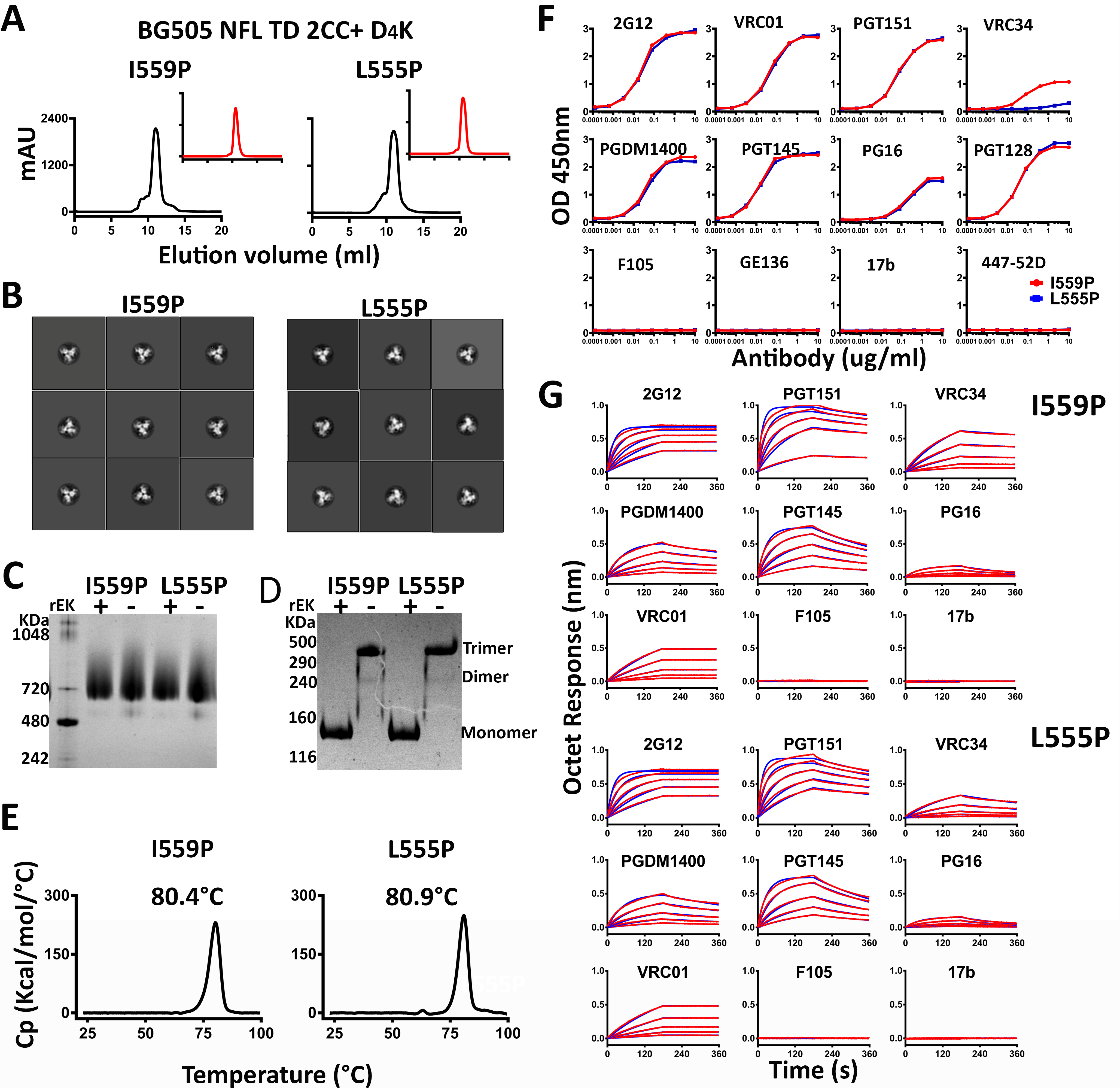

Supplement: Figure S6 — Characterization of BG505 NFL TD 2CC+ D4K I559P and L555P trimers. (A) size exclusion chromatography (SEC) profiles of BG505 NFL TD 2CC+ D4K I559P and L555P trimers following lectin-affinity purification. SEC profiles after F105 negative selection are shown in the insets. (B) 2D averages from negative stain electron microscopy of BG505 NFL TD 2CC+ D4K I559P and L555P trimers. (C) Blue-native PAGE analyses of BG505 NFL TD 2CC+ D4K I559P and L555P trimers with or without recombinant enterokinase (rEK) cleavage. (D) Disulfide bond formation was determined by SDS-PAGE under reducing and non-reducing conditions, respectively. (E) Differential scanning calorimetryr measurements of BG505 NFL TD 2CC+ D4K I559P and L555P trimers without rEK cleavage. (F) Comparison of ELISA binding properties of selected mAbs to BG505 NFL TD 2CC+ D4K I559P and L555P trimers without rEK cleavage. The EC50 values are summarized in Table 2. (G) BLI measurements of BG505 NFL TD 2CC+ D4K I559P and L555P trimers without cleavage. The fitting curves are shown in blue color and the kinetic parameters are summarized in Table 3. [file Image_6.tif]

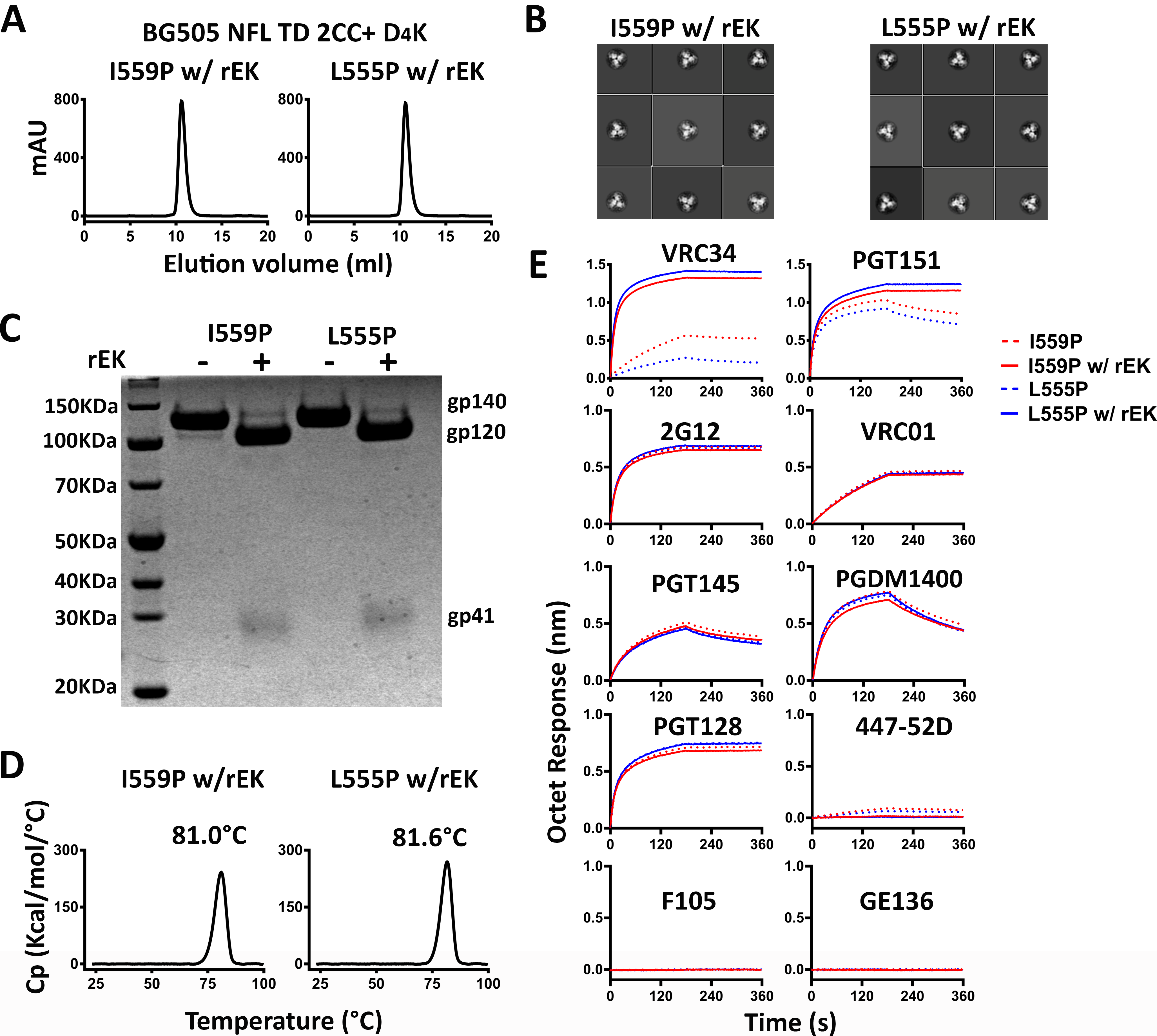

Supplement: Figure S7 — Characterization of BG505 NFL TD 2CC+ D4K I559P and L555P trimers after recombinant enterokinase (rEK) cleavage. (A) Size exclusion chromatography profiles of BG505 NFL TD 2CC+ D4K I559P and L555P trimers after rEK cleavage (w/ rEK). (B) Comparison of 2D averages from negative stain electron microscopy of BG505 NFL TD 2CC+ D4K I559P and L555P trimers after rEK cleavage. (C) Cleavage efficiency was determined by SDS-PAGE under reducing conditions. (D) Differential scanning calorimetry measurements of BG505 NFL TD 2CC+ D4K I559P and L555P trimers after rEK cleavage. (E) BLI measurements for BG505 NFL TD 2CC+ D4K I559P and L555P trimers interaction with selected mAbs before and after rEK cleavage. The kinetic parameters are summarized in Table 3. [file Image_7.tif]

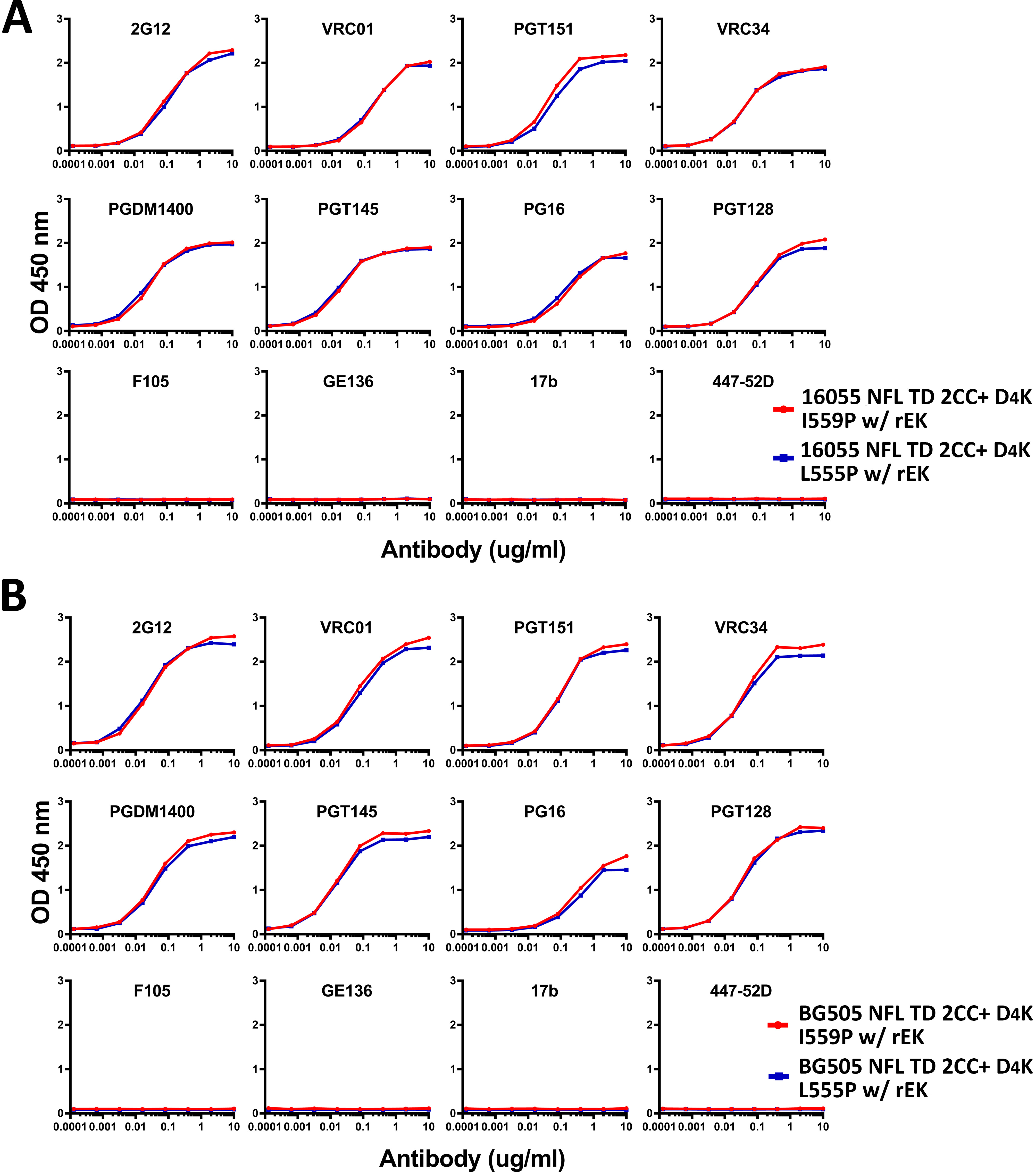

Supplement: Figure S8 — Comparison of ELISA binding reactivities between NFL TD 2CC+ D4K I559 and L555P trimers after recombinant enterokinase (rEK) cleavage. (A) Comparison of ELISA binding properties of selected mAbs to 16055 NFL TD 2CC+ D4K I559 and L555P trimers after rEK cleavage. (B) Comparison of ELISA binding properties of selected mAbs to BG505 NFL TD 2CC+ D4K I559 and L555P trimers after rEK cleavage. The EC50 values are summarized in Table 2. [file Image_8.tif]
