# Supplementary material for: Structure-Guided Redesign Improves NFL HIV Env Trimer Integrity and Identifies an Inter-Protomer Disulfide Permitting Post-Expression Cleavage
Source: Front Immunol. 2018 Jul 17;9:1631. doi: 10.3389/fimmu.2018.01631 (PMC6056610; doi:10.3389/fimmu.2018.01631)
Supplement: Supplementary file 9 [file Table_1.PDF]

Table S1. Cysteine linkage screening to stabilize soluble JRFL NFL trimers.

Name	Position #1	Region	Sec Stuc	Position #2	Region	Sec Stuc	Cα - Cα Distance (Å)	2G12	VRC06	PGT145	PGT151	F105	19b
	L34C	C1	S	W610C	CC-LOOP	L	5.51	+++ [a]	-	-	ND [b]	+	ND
	W35C	C1	S	V608C	CC-LOOP	L	5.62	+++	-	-	ND	+	ND
	W35C	C1	S	P609C	CC-LOOP	L	4.91	++	-	-	ND	+	ND
	T37C	C1	S	T605C	CC-LOOP	S	4.68	++	-	-	ND	++	ND
	Y39C	C1	S	L602C	CC-LOOP	L	5.62	++	-	-	ND	++	ND
	Y39C	C1	S	I603C	CC-LOOP	S	4.99	+++	-	-	ND	++	ND
	Y40C	C1	S	L602C	CC-LOOP	L	4.55	+++	-	-	ND	++	ND
	P43C	C1	L	A526C	FP	L	5.06	++	-	-	+/-	+	++
	N88C	C1	L	G527C	FP	L	4.62	++	-	-	+/-	+	++
SOS	A501C	C5	L	T605C	CC-LOOP	S	6.26	+++	++	++	+/-	+/-	++
	V89C	C1	L	G527C	FP	L	4.77	++	+/-	+	+/-	+	++
	T529C	FPPR	L	T627C	HR2	L	4.52	+++	-	-	-	++	++
	M626C	HR2	L	T529C	HR2	L	5.17	+/-	-	-	+/-	+/-	+/-
CC1	I201C	C2	S	A433C	C4	S	4.66	+++	++	+	+	+	+
CC2	A501C	C5	L	L663C	HR2	H	4.35	++	++	++	+/-	+	++

[a] Antibody binding is scaled from no binding reactivity (-) to strong binding (+++).

[b] ND: Not determined.
